# CIME4R: Exploring iterative, AI-guided chemical reaction optimization campaigns in their parameter space

**DOI:** 10.1186/s13321-024-00840-1

**Published:** 2024-05-10

**Authors:** Christina Humer, Rachel Nicholls, Henry Heberle, Moritz Heckmann, Michael Pühringer, Thomas Wolf, Maximilian Lübbesmeyer, Julian Heinrich, Julius Hillenbrand, Giulio Volpin, Marc Streit

**Affiliations:** 1 Johannes Kepler University Linz, Linz, 4040 Austria; 2 Division Crop Science, Bayer AG, Monheim am Rhein, 40789 Germany; 3 datavisyn GmbH, Linz, 4040 Austria; 4 Division Crop Science, Bayer AG, Frankfurt, 65926 Germany; 5 Division Pharmaceuticals, Bayer AG, Wuppertal, 42096 Germany

**Keywords:** Reaction optimization, Explainable AI, Artificial intelligence, Bayesian optimization, Interpretable

## Abstract

**Abstract:**

Chemical reaction optimization (RO) is an iterative process that results in large, high-dimensional datasets. Current tools allow for only limited analysis and understanding of parameter spaces, making it hard for scientists to review or follow changes throughout the process. With the recent emergence of using artificial intelligence (AI) models to aid RO, another level of complexity has been added. Helping to assess the quality of a model’s prediction and understand its decision is critical to supporting human-AI collaboration and trust calibration. To address this, we propose CIME4R—an open-source interactive web application for analyzing RO data and AI predictions. CIME4R supports users in *(i)* comprehending a reaction parameter space, *(ii)* investigating how an RO process developed over iterations, *(iii)* identifying critical factors of a reaction, and *(iv)* understanding model predictions. This facilitates making informed decisions during the RO process and helps users to review a completed RO process, especially in AI-guided RO. CIME4R aids decision-making through the interaction between humans and AI by combining the strengths of expert experience and high computational precision. We developed and tested CIME4R with domain experts and verified its usefulness in three case studies. Using CIME4R the experts were able to produce valuable insights from past RO campaigns and to make informed decisions on which experiments to perform next. We believe that CIME4R is the beginning of an open-source community project with the potential to improve the workflow of scientists working in the reaction optimization domain.

**Scientific contribution:**

To the best of our knowledge, CIME4R is the first open-source interactive web application tailored to the peculiar analysis requirements of reaction optimization (RO) campaigns. Due to the growing use of AI in RO, we developed CIME4R with a special focus on facilitating human-AI collaboration and understanding of AI models. We developed and evaluated CIME4R in collaboration with domain experts to verify its practical usefulness.

**Supplementary Information:**

The online version contains supplementary material available at 10.1186/s13321-024-00840-1.

## Introduction

Chemical reaction optimization (RO) plays a crucial role in every research and development endeavor in the domain of synthetic organic chemistry. The overarching optimization goals for a single chemical reaction are to use resources more efficiently, to reduce waste, and to increase the yield of the desired reaction product.

The fundamental challenge of RO lies in its expansive search space, which consists of a multitude of categorical (e.g., choice of reagent, base, catalyst, methods) and continuous (e.g., temperature, concentration, reagent equivalent) parameters. Some common approaches [1] to solving such a task are high-throughput experimentation (HTE) [2], one factor at a time (OFAT) [3], design of experiment (DoE) [4], and, more recently, AI-guided optimization [5–7]. All approaches share the ultimate goal of identifying one or more optimal solutions within the high-dimensional parameter space.

The absence of suitable visualization tools makes comprehending the parameter space difficult, leaving ambiguities regarding explored domains, actual reaction performance, and anticipated outcomes. In the case of AI-guided RO, an additional level of complexity is added because the AI’s data (e.g., predicted values, uncertainty of predictions, or feature importance of a prediction) is often difficult for humans to grasp without any additional tools to explore it. This makes it hard for scientists to understand how a model arrived at a particular prediction [8], which is important to detect possible flaws and shortcomings of the AI model and to calibrate user trust [9]. Understanding an AI prediction could also help scientists to learn from a model if they deem the decision to be reasonable, thus enhancing their scientific understanding [10].

Static Explainable Artificial Intelligence (XAI) techniques can support users in understanding AI models [11–13], but interactive tools are needed to aid users in exploring and understanding complex models [14–16]. Employing visualizations and visual analytics tools can aid humans in understanding complex datasets in a variety of domains [15, 17–19]. Currently, many scientists fall back on using spreadsheets as their main analysis tool. Although there is little overhead to learning how to use spreadsheet tools, they are suited mainly to simple analysis tasks, such as sorting or filtering by some value of interest. More elaborate general-purpose dashboard tools, such as Spotfire [20], enable users to interactively explore their datasets [21]. Off-the-shelf tools also exist that implement basic chemically-aware functionalities, like molecule structure rendering or substructure search [22], and commercial chemical analytics tools [23]. However, these off-the-shelf tools lack the ability to accommodate the challenging aspects of RO datasets, such as large parameter spaces that contain a mix of static and temporal data and data uncertainty arising both from limited data availability for the model and uncertainty and error in the measured data points. (see Sect. 1.2 for more details). Similarities to prior work [24–28] on visual analysis of parameter spaces in other domains exist, but these approaches are not directly applicable to RO data and its challenges.

### RO data and workflow

**Fig. 1:**
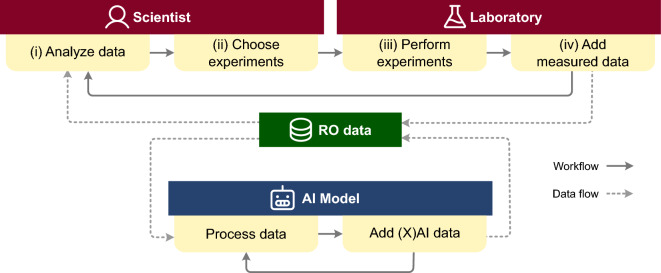
Outline of an iterative RO workflow. Scientists analyze RO data (i) and choose a batch of experiments to be performed (ii). The experiments are carried out in the lab (iii), and RO data is updated with the measurements from the experiments (iv). In a follow-up step, scientists analyze the updated data (i), and a new batch of experiments is selected (ii). The RO dataset can be augmented with data from AI models (blue). CIME4R aids the analysis of RO data and facilitates informed decision-making

A dataset from RO workflows can consist of the following variables: *parameters*, *lab measurements*, *metadata*, *AI predictions*, and *AI explanations*. A typical RO workflow starts with a scientist deciding on the *parameters* (e.g., temperature, base, solvent) they use to optimize one or more target values (e.g., yield, selectivity, side components). The pool of all possible combinations of parameter values defines the parameter space and forms the basis of an RO dataset. Each combination of parameter values defines one specific experiment that can be performed to obtain *lab measurements*. Scientists can add additional *metadata* to this dataset, for example, molecular descriptors [29] of chemical components that can be used in the RO campaign. The RO workflow varies depending on the method chosen for the optimization campaign. Fig. 1 illustrates a typical workflow for iterative approaches. It usually starts with scientists (i) analyzing the RO data and (ii) choosing a batch of initial experiments, which are then (iii) performed in the laboratory. After the experiments have been completed, (iv) the RO dataset is augmented with the measurement data, for example, the measured yield or number of the cycle in which the experiment was performed. Scientists then (i) analyze the updated dataset to (ii) decide on the next batch of experiments. The process is repeated until a satisfactory outcome is reached (e.g., the yield is sufficient). To illustrate the AI-guided workflow in addition to the scientist-driven workflow, we consider an additional feedback loop where the RO dataset is given to an AI for processing (see Fig. 1, bottom). For example, for EDBO (Experimental Design via Bayesian Optimization) [5] the RO dataset can be augmented with the *AI predictions* for each possible experiment and the corresponding uncertainty with which the prediction was made (e.g., predicted yield and variance). These predicted values are fed into a so-called acquisition function to decide which batch of experiments to perform next. In addition to prediction outcomes, XAI methods can be used to add any kind of *AI explanations* that might be helpful to understand the optimization process (e.g., SHAP values tell us which parameters of an experiment were important for the prediction).

From looking at this workflow, it becomes clear that the RO dataset becomes increasingly complex with every optimization iteration. In this work, we propose CIME4R, a novel visual analytics tool that aids scientists in analyzing RO data. It helps scientists to make informed decisions during the RO campaign and to review RO campaigns in retrospect, especially in the realm of AI-guided RO. CIME4R is open-source to allow the community to enhance the tool and share their input.

### User tasks and challenges

We identified two points in time at which scientists require analysis of RO optimization data: *during* and *after* the optimization process. *During* RO, scientists have to decide on the next batch of experiments based on all the data from the current and previous cycles. Scientists must be able to balance exploration (i.e., collecting new information about unseen regions) and exploitation (i.e., optimizing within a known, high-performing region) of the parameter space, as otherwise the RO campaign is prone to stopping at a local maximum without having considered most of the reaction space. With AI-driven RO, exploration and exploitation are balanced implicitly with the use of acquisition functions. However, users of AI models have to understand the predictions of their models such that they can judge their actual performance and shortcomings [9]. Only then can users decide whether it is better to follow an AI’s suggestion or to overrule it and rely on their own experience. In summary, scientists should be able to understand the RO campaign to make informed decisions. For AI-guided RO, scientists should be able to effectively combine their own chemical intuition with an AI model’s computations to make an informed decision on the next batch of experiments. *After* the RO campaign, scientists want to learn from the collected RO data to gain insights for future RO campaigns. In scenarios where unsatisfactory results have been reached, scientists need to be able to identify the problem with their own or an AI’s decision. If the results are satisfactory, chemists can learn from their colleagues or the AI model’s decisions and improve their own understanding and intuition. Therefore, scientists should be able to effectively review the entire RO campaign to see whether the problem was approached in a useful way or whether bias in decision-making led to a suboptimal solution.

We identified four main analysis tasks **(T)** and their associated challenges **(C)** that are critical for scientists to achieve these goals and that guided the development of CIME4R:

***(T1) Comprehend a parameter space:*** Gain an overview of the extent of an RO parameter space. See which experiments have already been explored and which have not. Gain an overview of an AI model’s predictions of the parameter space. See whether a model focuses on exploring unseen regions of a parameter space or whether it maximizes outcomes within known high-performing areas.

***(C1) Exponentially growing parameter space:*** RO datasets grow exponentially with with the number of parameters that are optimized. For example, if we seek to optimize temperature, base, and solvent, and for each of these parameters we define five values for optimization, the whole combinatorial parameter space is $5*5*5=5^3=125$; if we then choose to optimize for 10 different reagents, the parameter space grows to 1250 different experiments. This exponential growth soon becomes a challenge from computational resource and human perception perspectives.

***(T2) Investigate optimization progression:*** Understand how data changes over the optimization cycles. Temporal variables are measurements—or calculations based on such measurements—that are accumulated throughout the optimization process. For example, after each cycle, we add new values for measured yield to the dataset. We can also add the model predictions that are based on measurement results and are therefore updated after every cycle.

***(C2) Visualizing temporal data:*** The time series information introduced adds a new layer of complexity to the dataset. Visualizing a mix of static and temporal data is not straightforward and requires careful consideration in the design process.

***(T3) Identify critical factors:*** Identify which (combinations of) parameter values are predictors for a high/low target value (e.g., yield). Identify which parameters an AI model deems important for predicting a high/low target value.

***(C3) Interaction of parameter values:*** Sometimes, particular parameter values have a positive or negative effect on an objective (e.g., for a particular reaction, high temperature may always give a better or low temperature may always give a lower yield). However, typically, parameters interact with each other and only particular combinations of parameter values give good results. For example, low temperature results in a high yield only when combined with a specific substrate, and in a lower yield with any other reagent. Determining these correlations is an essential part of understanding the optimization problem and the parameters used.

***(T4) Understand model predictions:*** Understand which parameters were most important for an AI model’s prediction and whether this information overlaps with an expert’s judgment. Detect and overcome possible flaws and shortcomings of AI models to make informed decisions on the next batch of experiments. Learn from AI reasoning and enhance expert knowledge about reaction parameters.

***(C4) Data uncertainty:*** The RO campaign may start with no prior measurement data or direct knowledge of the reaction to be optimized. Only after the first cycle of experiments (randomly chosen or chosen by experts) do we have a small set of measurement data that is used to model the whole parameter space. Estimations of a new experiment that is close to those already performed can be made with higher confidence than experiments that fall within an unexplored area. The optimization process is usually a trade-off between *exploring* uncertain areas of the parameter space and *exploiting* slight variations in the parameter values of experiments that previously performed well. It is therefore important to illustrate the estimation of a value together with the (un)certainty of the estimation [30, 31].

## Design

CIME4R extends CIME [15], a system developed for exploring and comparing Explainable Artificial Intelligence (XAI) outputs from chemical AI models. In contrast to CIME, CIME4R was developed for analyzing RO datasets, supporting scientists in their decision-making, and making AI-guided RO better understandable. It visualizes large and high-dimensional parameter spaces and is able to handle iterative and uncertain data, which is essential for analyzing RO data. CIME4R was developed by visualization experts in close collaboration with chemists. Throughout design and implementation, we continuously tested CIME4R in terms of its usability and usefulness for the intended tasks. For testing we chose a subset of the deoxyfluorination dataset with results from EDBO by Shields et al. [5]. Finally, we confirmed the usefulness of CIME4R using two different datasets, as described in Sect. 4. All datasets and their creation scripts are available online [32]. In the following subsections, we elaborate on the design choices we made to support users in executing their tasks (as defined in Sect. 1.2) to meet their RO goals.

### Data filtering

**Fig. 2:**
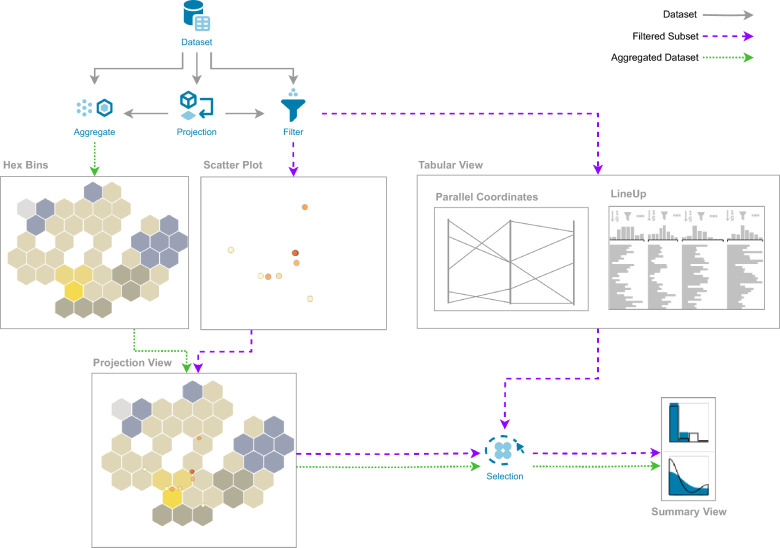
Overview of CIME4R’s main components and the type of data that flows through each component. We differentiate between using the entire dataset (solid grey arrow), an aggregation of the dataset (green dashed arrow), and a filtered subset of the dataset (purple dashed arrow)

To handle large datasets **(C1)**, we decided to implement filtering and aggregation strategies. We use filtering to allow users to choose a meaningful subset of the parameter space that is relevant to their analysis. Most visualizations are based on the filtered data subset. However, to gain a better understanding of the entire dataset, CIME4R provides additional visualizations that show aggregations of the full data. Figure 2 gives an overview of which visualizations and computations operate on a filtered subset, an aggregation of the dataset, or the whole dataset.

In addition to the filtering functionality, we allow users to define exceptions to filters. In other words, although a set of experiments is not included within the specified filter, an exception can be made to include them. This was implemented to help users to analyze data in more detail, for example, when users want to explore a particular area of the parameter space in more detail without changing the remaining filtering.

### Projection view

We utilize dimensionality reduction (DR) to gain an overview and be able to comprehend a parameter space **(T1)**. This generates a two-dimensional space that is visualized in the projection view [15]. In addition to choosing from UMAP^1^, t-SNE^2^, or PCA^3^ as DR techniques, users can specify which features are more or less important than others by applying weights to the variables. By exploring a variety of weightings, users can analyze the parameter space under various perspectives and pick the one that best facilitates the analysis (see Supplementary Material, Additional file 1 for details).

**Fig. 3:**
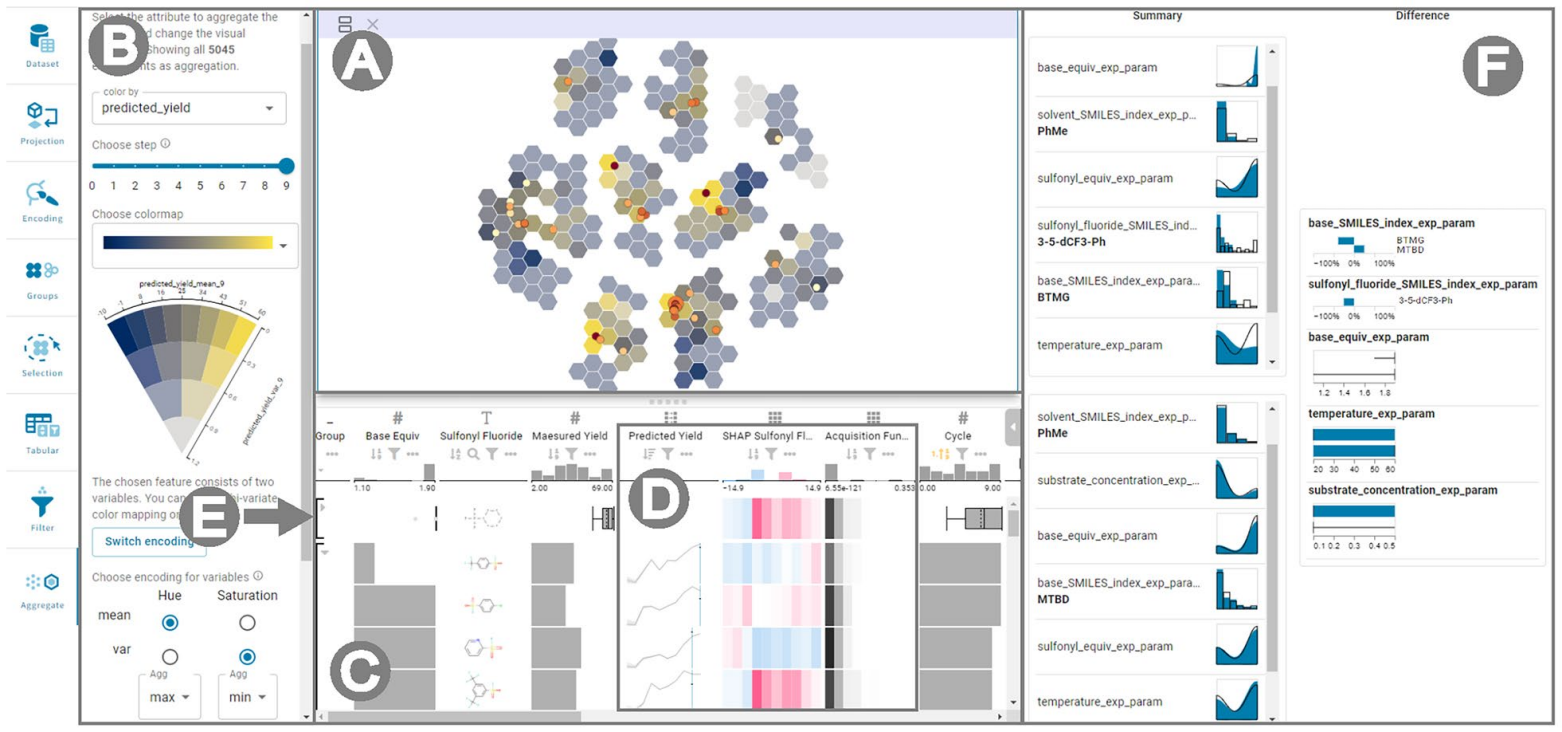
The CIME4R’s user interface showing the (**A**) projection view, which gives an overview of the reaction-parameter space, (**B**) aggregation settings side panel, where users can adjust the visual encodings of the hexagonal bins in the projection view, (**C**) tabular view with LineUp, which gives details about each experiment, (**D**) temporal columns and (**E**) summaries in LineUp, and (**F**) summary view, which shows similarities and differences of sets of experiments. This example shows the results of Bayesian RO of a deoxyfluorination reaction examined by Shields et al. [5]

CIME4R includes two views that show this two-dimensional projection of the parameter space (see Fig. 3A): (*i*) A scatter plot visualizing the filtered subset, where each experiment corresponds to one point; and (*ii*) an aggregated view of the entire parameter space (i.e., all possible reaction combinations), which is shown as colored hexagonal bins in the background of the projection view. While experimenting with a range of techniques to analyze the aggregated two-dimensional space, we tried to visualize the data as a continuous heatmap and used interpolated values for areas without data points, similar to the landscapes shown in other work [5, 7, 35] (see Additional file 1 for details). However, such a visualization suggests both the presence of data (i.e., we do not actually have values in some areas, but fill the space with calculated values) and continuity of data (i.e., assuming that there is a continuous change between experiments in a parameter space) where they do not exist, which might mislead users. After careful consideration, we chose discrete bins to show the aggregated values. We decided to use hexagonal bins as a trade-off between squares, which are space-filling, and circles, which have borders with a uniform distance to the center [36, 37]. To give the aggregated view more flexibility, we implemented the hex bins to dynamically adapt to the zoom level. This means that users gain an abstract overview at first, but when zooming in the hex bins become more fine-grained, making the view more detailed (see Additional file 1 for details). Overall, the projection view gives users an overview of the entire parameter space of the dataset in two dimensions and a more detailed view of the filtered subset, helping them to comprehend a parameter space **(T1)**.

The visual encoding of points in the scatter plot and hexagons in the aggregation can be changed to better comprehend a parameter space **(T1)** in the two-dimensional space and identify critical factors **(T3)**. For example, users can choose to encode the size of points in the scatter plot by temperature or choose a color encoding to distinguish between high and low yields. The aggregation hexagons can, for example, be colored by the value of the acquisition function to see which area in the parameter space the model deems best to be explored next. This functionality can also be used to understand model predictions **(T4)**.

To analyze temporal data **(C2)** in the projection view, we integrated a slider that allows users to easily navigate step-wise through time and see how the values in the parameter space change with each cycle (see Fig. 3B). Animating over time steps or switching between time steps helps to gain an overview of the changes happening in the parameter space. However, showing changes consecutively may raise the problem of change blindness, where small but possibly important changes escape users’ perception [38]. For a simultaneous view of one or several time steps, we developed the option of juxtaposing multiple projection views (see Additional file 1 for details). This allows users to compare in more detail how values change with each cycle and to investigate iterative data **(T2)**.

Finally, to show uncertain data **(C4)** in the hexagonal bins, we make use of bi-variate color mapping and VSUP (value-suppressing uncertainty pallets [30]). Bi-variate color mapping is a technique that combines two principles of color encoding to represent two variables simultaneously (i.e., one variable is mapped as a variation of hue and the second as varying saturation, which results in a 2-d matrix of a mix of hues and saturation). As an alternative to the standard bi-variate color mapping, we chose to also implement VSUP, which is particularly useful if data contains uncertainty. A VSUP color map is represented in the form of a wedge, as shown in Fig. 3B. The layers of the wedge represent the uncertainty of the data (i.e., the further from the center, the lower the uncertainty). The rationale behind this design choice is that values that are uncertain do not have to be as distinguishable from each other as those with high certainty. For example, a model predicts the yield for every possible experiment; the variance/uncertainty depends on the data already available in a specific region of the parameter space. High variance of the model’s prediction indicates that this part of the parameter space has not yet been adequately explored and insufficient data points are available to model it well, which makes the actual predicted value less important. The advantage that comes with VSUP is that values with higher certainty are represented with a higher number of distinct hues, and values with lower certainty have fewer bins. This helps users to utilize and understand model predictions **(T4)**.

As an alternative to bi-variate color mapping and VSUP, uncertainties could also be explicitly encoded in a second visual channel [30] (e.g., visualizing the value as face color and the uncertainty edge color of the hexagons). However, separate encoding of value and uncertainty increases the complexity of a visualization and users’ cognitive load. If users want to see the values and uncertainties anyway, parallel views can be used to show the data separately.

### Tabular view

For a detailed view of the parameter space, we decided to include a LineUp table [39]—an interactive visualization for tabular data—and modified it to make it suitable for reaction optimization data (see Fig. 3C). The LineUp table allows users to view the parameters and values (columns) for each experiment (rows) that is shown in the scatter plot, enabling users to see more detailed information about each experiment. Its interactive features let users select, sort, filter, rank, or group by columns to explore the data.

To visualize temporal data **(C2)** in LineUp, we accumulate the temporal columns that belong together and show them in a single one-dimensional heatmap (see Fig. 3D). For example, all values of the acquisition function for each cycle are visualized in one heatmap that changes colors corresponding to the timesteps along the x-axis^4^. The progression over time is thus visualized in a compact way and users can investigate iterative data **(T2)**.

Since LineUp does not support simultaneous representation of temporal data **(C2)** and uncertain data **(C4)**, we developed a custom LineUp column that shows the mean predicted value after each cycle as an interactive line chart and the uncertainty of the prediction as an area around the line (see Fig. 3D). This column helps users to understand how a model’s prediction developed over time and to understand model predictions **(T4)**.

**Fig. 4:**
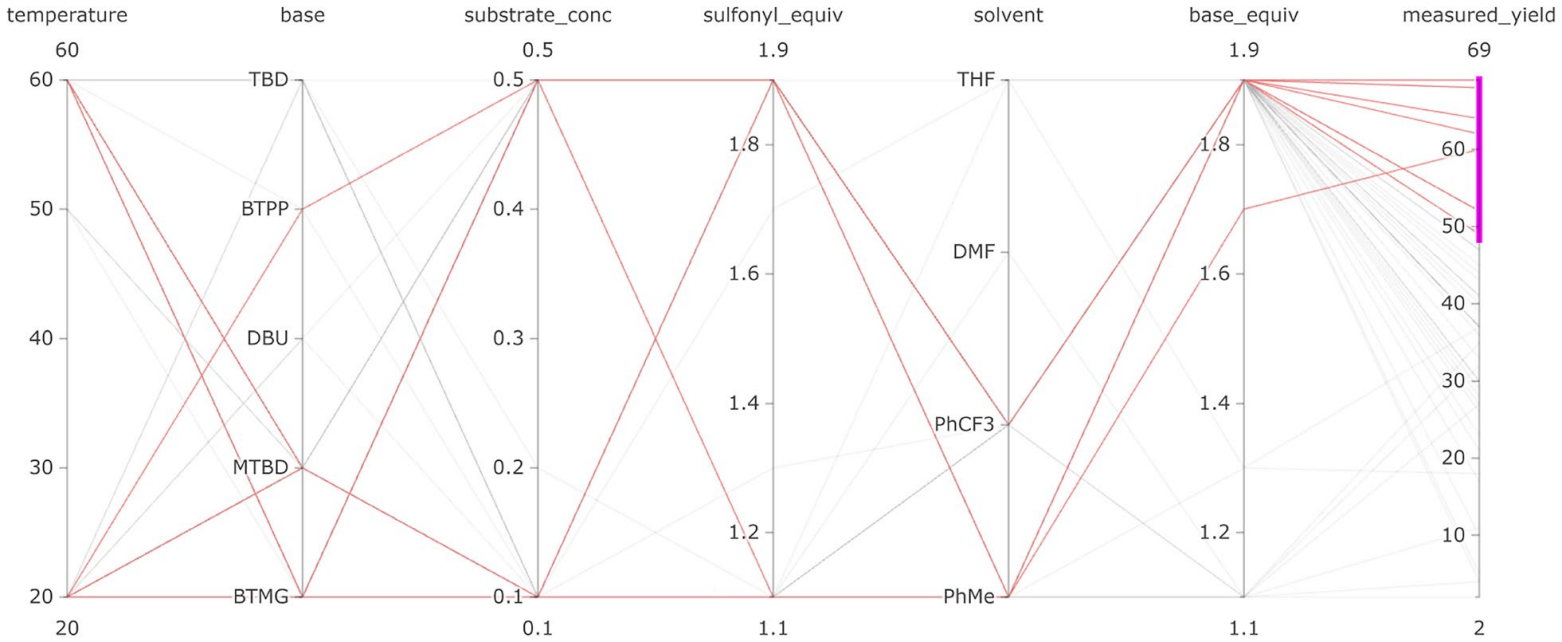
Users can open a parallel coordinates plot that replaces the LineUp table to explore the interactions between parameter values and identify critical factors in an optimization process. Each line represents the conditions of one reaction, lines highlighted in red represent reactions that have been manually selected due to having a high yield (purple selection)

To identify critical factors **(T3)**, users can use LineUp’s aggregation functionality to view summaries for groups of experiments (see Fig. 3E). For example, users can identify the top 10 experiments in terms of predicted yield. They can then compare the summaries and find which parameter values might result in a high predicted yield.

For analyzing the interaction of parameter values **(C3)**, we implemented a parallel coordinates view. Users can interactively select parameter values of interest, and the parallel coordinates plot highlights the correlations between the parameter values, as shown in Fig. 4. Users can thus determine which parameters or combinations of parameters predict a higher/lower yield, which makes it easy to identify critical factors **(T3)**.

Both tabular views are linked to the projection view via selection. This means that users can select experiments in one view, and the corresponding points are automatically highlighted in the other view, which combines the strengths of both views in an interactive way.

### Summary view

The summary visualization shows the distributions of feature values for selected experiments and compares them to the distributions of the remaining data (see Fig. 3F) [18]. This can be done not only for the filtered subset, but also for the entire dataset to support handling of large datasets **(C1)**. By selecting the clusters formed during the projection, users can explore the projection space to comprehend a parameter space **(T1)** and form an intuition. They can also inspect experiments to see which parameter values, or combinations thereof, predict a high yield and thus identify critical factors **(T3)**.

To inspect the interaction of parameter values **(C3)**, users can see the differences between groups of experiments (e.g., experiments with high yield vs. experiments with low yield) in the group comparison view. This view shows the summary visualization for each of the groups that are compared, and additionally a visualization that explicitly shows the difference between two groups. For example, in Fig. 3F, when comparing the group of high-yielding experiments with the group of low-yielding experiments, the base and sulfonyl fluoride compounds and base equivalents differ most between the two groups. To keep the visualization simple, only the feature values that have large changes between the two sets are displayed (e.g., for the base, only the changes of BTMG and MTBD compounds are displayed). The group comparison helps users to see the feature values that differ most between two groups, making it easy to find well-performing parameter combinations to identify critical factors **(T3)**.

The group comparison view can also be used to investigate iterative data **(T2)**. More concretely, users can compare the experiments chosen for each cycle and see the differences between the cycles. This aids users in understanding how the decision-making evolved over the whole iterative process of RO.

## Implementation

The front end of CIME4R is a React [40] app and uses the Projection Space Explorer [41] (PSE) library. This library provides a general layout for interactive web applications that utilize projections to explore a data space. The individual components of the app can be customized to specific use cases. In the course of developing CIME4R, we modified the PSE library to make it more flexible and accommodate further customization of its features and components.

The back end server is a python application that uses Flask [42] to serve the API. For molecule computations and rendering, we used the RDKit [43] python library. We added a back end component to allow handling of large datasets **(C1)**, which come with RO tasks. To cope with these large datasets **(C1)** and avoid memory issues, the data has to be either (*i*) processed in chunks of smaller pieces of data, (*ii*) filtered to a subset of the whole dataset, or (*iii*) aggregated to reduce the overall size of the dataset. All of these optimizations are done in the back end after the dataset has been uploaded. Figure 2 gives an overview of which CIME4R components use the whole dataset, aggregations of it, or a filtered subset.

### Data processing

Datasets must be provided in CSV format (comma-separated values), where each row corresponds to one particular combination of parameter values (i.e., one possible experiment). In addition to the parameter values, other relevant data can be included for each experiment: measured target values if available for this experiment (e.g., measured yield); the cycle in which the measurement was performed; the predicted target value for each cycle (e.g., predicted yield of an experiment); explanations for model predictions (e.g., SHAP [13] values that indicate which parameter values were important for this prediction); and any other information that might be of interest to users.

Columns can be named arbitrarily; however, some special column names and modifiers are recognized by the back end and used during processing. This helps with customizing the treatment of some variables.^5^ Examples of dataset creation and a description of all modifiers are linked directly in CIME4R and the official user documentation [44].

After uploading a dataset to the back end,^6^ the data is saved in chunks to a PostgreSQL [45] database for easier and faster access in future requests. When the dataset is saved, the front end can request a filtered subset or aggregations of the dataset from the back end to use for the visualizations.

### Projecting data

While users can choose the type of dimensionality reduction (i.e., UMAP [33], t-SNE [34], and PCA) and adjust the projection parameters in the UI, the computations are done in the back end. Performing the computations in the back end allows us to project the entire dataset and not just the subset displayed in the front end. For large datasets, it is computationally unfeasible to project the entire data at once. In these cases, we first reduce the number of features with an incremental version of PCA that processes the data in chunks. The resulting lower-dimensional features can then be used as input to UMAP or t-SNE. For a comparison of projections with and without chunking, see Additional file 1. Users can choose between Euclidean (for numerical data), Jaccard (for categorical data), and Gower [46] (for mixed data types) as distance metrics for the projection. Since Gower’s distance is not included in the python libraries that we used for dimensionality reduction, we implemented it as a function that produces a pre-computed similarity matrix. Similarly, to enable the assignment of custom weights to features, the similarities must be pre-computed. Occasionally, the projected space results in overlapping points. We added an overlap removal method to reduce overlaps in the scatter plot [47].

The two-dimensional space is visualized in the front end using a scatter plot for the filtered subset of the data and hexagonal bins for an aggregated view of the entire dataset.

### Aggregating data

We modified the PSE library to accept additional custom layers in the projection view that are rendered above or below the scatter plot layer. Thus, we were able to implement a layer that shows an aggregation of the entire dataset using hexagonal bins as the background of the scatter plot. To compute the aggregations, we implemented a function that determines whether the coordinates of a point are contained in the hexagon at a certain position. All values that are contained in a particular hexagon are then aggregated by a user-defined aggregation function (i.e., min, max, median, mean, or count). All computations are done in the back end, and only the aggregated values and the coordinates of the hexagon centers are served to the front end.

## Results

In this section, we describe the results of three case studies. These were conducted by four collaborators (three of them authors), who are experts in the field of chemical RO. We prepared two datasets [32] to be analyzed by the experts, who then used CIME4R for *(i)* understanding and learning from RO campaigns in retrospect (with and without AI-generated data), and *(ii)* human–AI collaborative decision-making. All experts performed their analyses independently of each other. The following sections summarize the most important insights and show how CIME4R can be utilized.

### Case study 1: Buchwald-Hartwig reaction—learning from and understanding a fully measured RO space

**Fig. 5:**
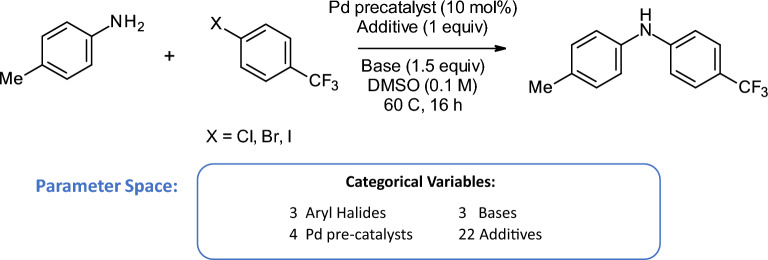
Reaction scheme for the Buchwald-Hartwig reaction used as the dataset for case study 1 [48][5]

**Fig. 6:**
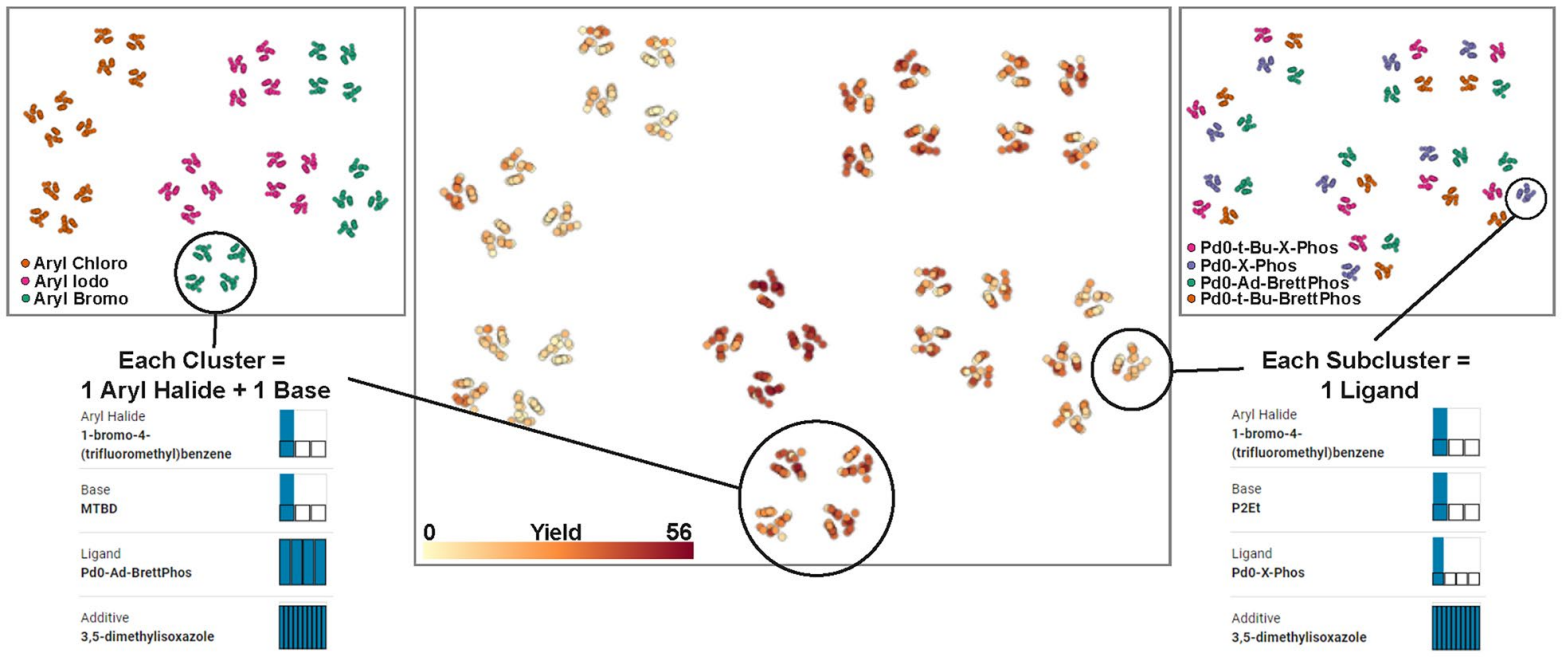
Parallel projection views of the Buchwald-Hartwig reaction space. Each parallel view shows the same projection of the parameter space, but with a different color encoding. The left projection view encodes the three compounds of aryl halide. The view in the center visualizes the measured yield, and the right projection encodes the four ligand compounds. The chemists used the projection view in combination with the summary view—which shows summary statistics of currently selected experiments—to comprehend the parameter space and the clusters formed during the projection **(T1)**

In the first case study, we asked the chemists to use CIME4R to understand a reaction space that had been fully tested experimentally. We chose a subset of a Buchwald-Hartwig reaction dataset performed and reported by Ahneman et. al. [48]. As shown in Fig. 5, this reaction involves four categories of compounds (three aryl halide substrates, three bases, four ligands, and 22 additives), with a sample of 792 experiments performed. We visualized the experiments in CIME4R by means of a weighted t-SNE projection using the experiment parameters and their associated descriptors (see Additional file 1 for details).

Using the selection and summary tools available in CIME4R, the chemists were able to quickly identify and understand the clusters of experiments in the projection view **(T1)**, as shown in Fig. 6. Each of the nine main clusters represents the combination of one aryl halide and one base. Within each cluster, four sub-clusters exist, each of which represents the use of one ligand, and the remaining points within each sub-cluster represent the various additives used.

**Fig. 7:**
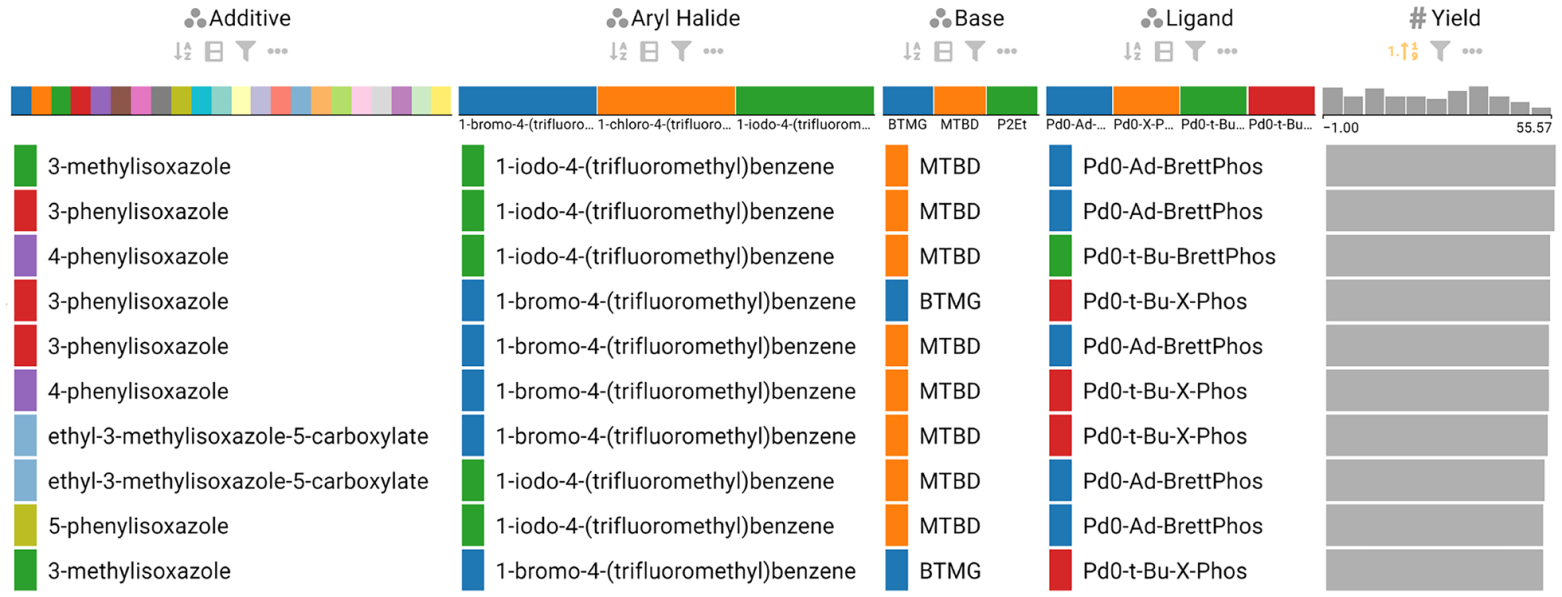
LineUp visualization of the 10 best-performing reaction conditions, sorted from highest to lowest by measured yield. Users can determine which factors of a reaction contribute positively to the yield **(T3)**

By color-encoding each point in the scatter plot with the measured yield, the chemists were able to easily identify that lower yields were achieved with the aryl chloride substrate than with the aryl bromide and iodide substrates. To identify the reactions with the best conditions, they used LineUp and ordered the experiments by the measured yield, as shown in Fig. 7. Here they observed that the aryl iodide substrate with MTBD as a base and Pd0-Ad-BrettPhos as the catalyst gave the best yield, with multiple options for the additives providing a high yield **(T3)**.

**Fig. 8:**
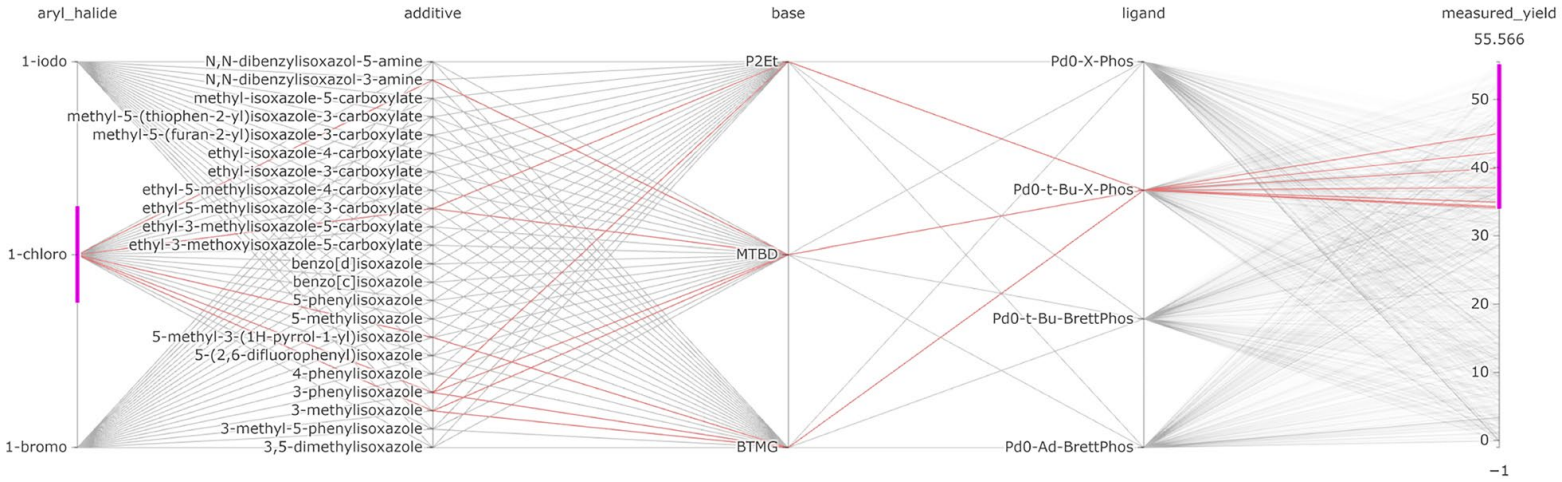
Parallel coordinates visualization of the Buchwald-Hartwig reaction filtered to show the reaction conditions that give high yield when using the aryl chloride substrate (highlighted red lines) **(T3)**

Although the aryl chloride gave a lower yield on average, it may be interesting to determine what conditions are successful for this substrate; for example, the aryl chloride substrate may be more favorable for cost or availability reasons. Therefore, the chemists looked into which suitable reaction conditions are able to transform the aryl chloride substrate into the desired product. Using the parallel coordinate visualization (see Fig. 8), the chemists observed that for a moderately high yield, Pd0-t-Bu-C-Phos was the best-performing ligand and performed well with all bases. This was in contrast to the aryl iodide substrate, which gave high yields with three possible ligands, but not with P_2_Et as the base.

### Case study 2: direct arylation reaction—learning from and understanding an RO campaign in retrospect

**Fig. 9:**
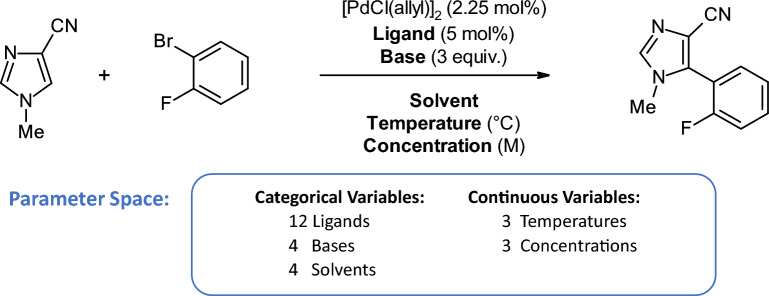
Reaction scheme for the direct arylation reaction used as the dataset for case studies 2 and 3 [5]

**Fig. 10:**
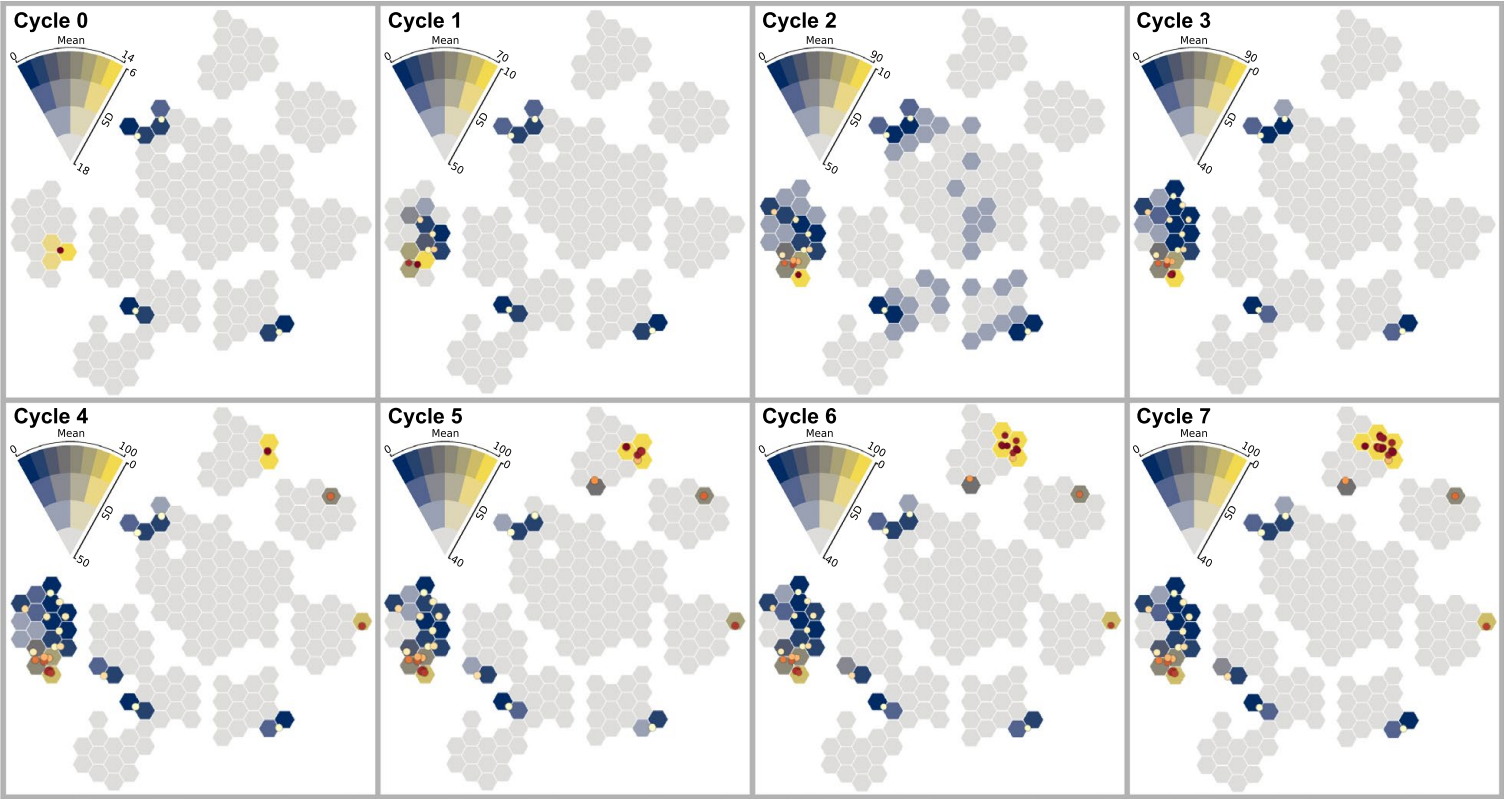
Projection of parameter space, aggregated by predicted yield, and standard deviation showing the progression of EDBO after each cycle **(T2)**. The projection view gives users an overview and helps users to comprehend the parameter space **(T1)**

**Fig. 11:**
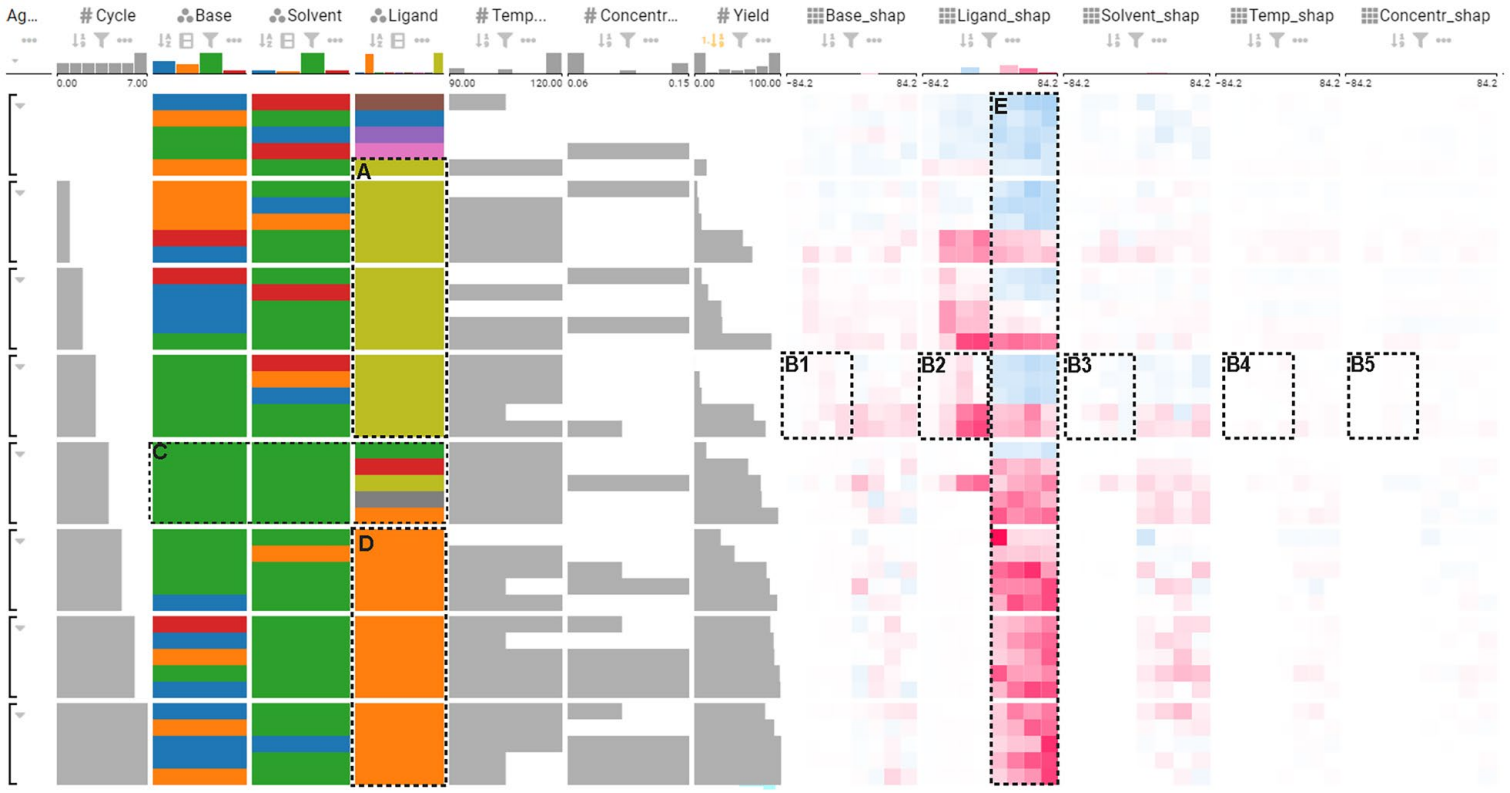
LineUp overview of the reactions performed, their measured yields, and calculated SHAP values, grouped by cycle (0–7) and ordered by measured yield. After a random initialization in cycle 0, EDBO prioritized experiments with the currently best ligand (**A**) for the next three cycles. In cycle 4, EDBO explored different ligands, but for fixed base and solvent (**C**). Finally, EDBO managed to find a yield of 100% by exploiting the best-performing yield found in cycle 4 (**D**). EDBO’s choice of parameters and the development of the SHAP values (**B1**–**5**, **E**) aid users in understanding the model’s decisions **(T4)**

In this case study, we asked the chemists to use CIME4R to explore and retrospectively understand a chemical RO campaign. We used the direct arylation optimization that was performed by Shields et al. [5], as shown in Fig. 9 with their proposed EDBO model [49]. This reaction involves three categories of compounds (12 ligands, four bases, and four solvents) and two numeric variables (three temperatures and three concentrations) resulting in a pool of 1728 experiments. The reaction was optimized with EDBO over eight iterations with five experiments per iteration. To prepare the data for CIME4R, we created a dataset that contained all possible experiments and—where the experiments had been performed—measurements. We then added the means and standard deviations of the yield predictions by EDBO and the corresponding values from the acquisition function for each cycle. Finally, using the Kernel SHAP method [13], we also calculated the SHAP values of the EDBO input features for each cycle. The code for creating this dataset is publicly available [32]. We visualized the experiments in CIME4R by means of a weighted t-SNE projection using the experiment parameters and SHAP values (see Additional file 1 for details). Figure 10 shows CIME4R’s projection view of the dataset **(T1)** and its development throughout the RO campaign **(T2)**.

**Fig. 12:**
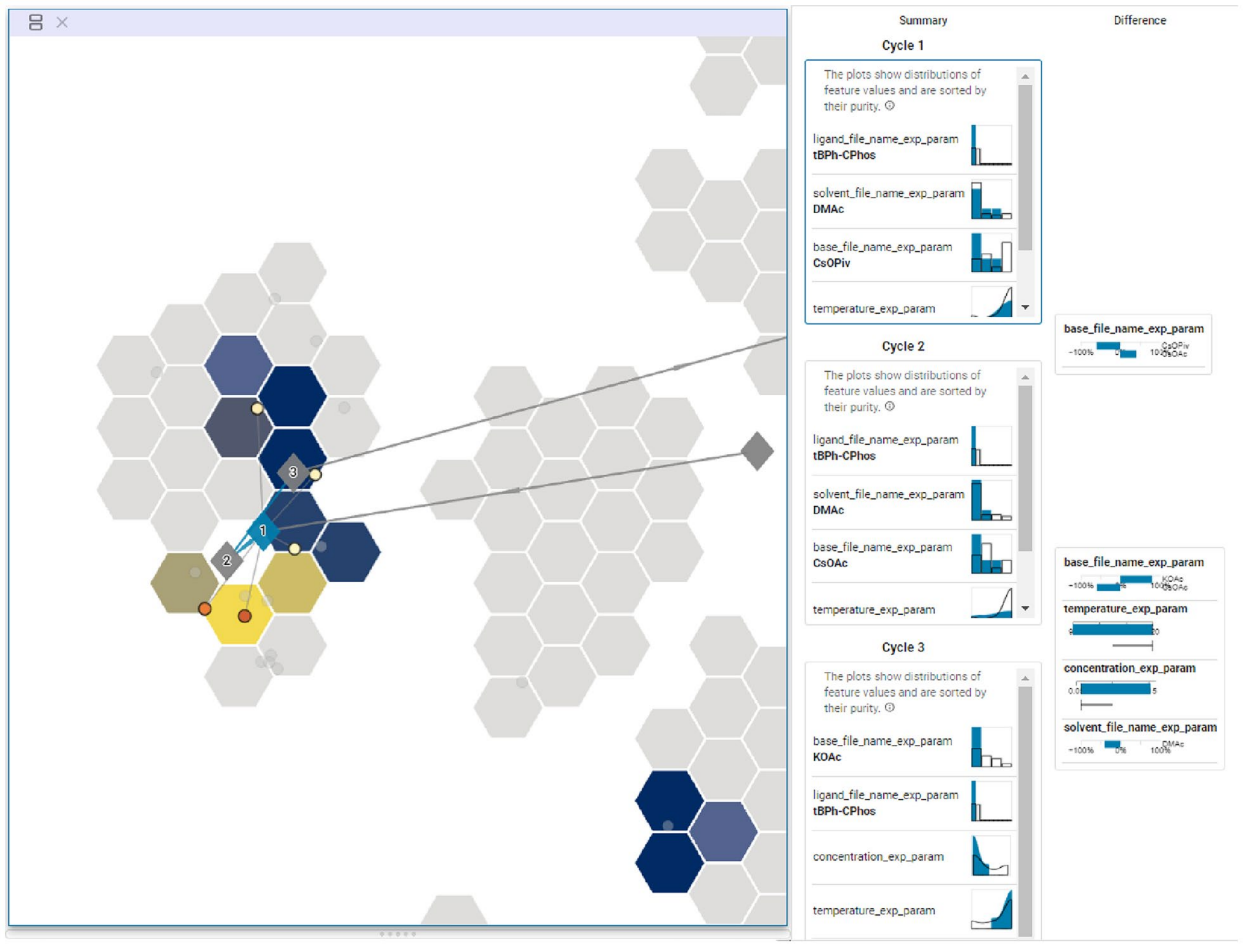
Projection and summary views of cycles 1–3 in case study 2, giving an overview of how EDBO progressed over these cycles **(T1)** **(T4)**. The diamonds are the centroids of the experiments for the corresponding cycles. For cycles 1–3, the centroids fall within a small region, which means that the model exploited the currently best solution to find the optimal solution. The summary visualizations give an overview of which parameter values the model explored during this cycle, while the difference visualizations show how the cycles differed from each other

While investigating this dataset in CIME4R, the chemists made several discoveries that helped them to understand the EDBO campaign **(T4)**. After performing five initial random experiments (cycle 0), the chemists discovered that EDBO prioritized experiments with the best-performing ligand up to this point (tBPh-CPhos) for the next three cycles. This development can be seen in the LineUp overview (see Fig. 11 A) and in the projection and summary views of the corresponding cycles (Fig. 12). The chemists found one cluster of potential experiments that contain the best conditions up to this cycle (i.e., the combination of a tBPh-CPhos ligand, DMAc solvent, and KOAc base), which resulted in experimental yields from 67 to 89%. The importance of ligand, solvent, and base to the current EDBO prediction was also indicated by the positive (pink) SHAP values seen in the first four cycles of the reaction (see Fig. 11 B1–B3). In contrast, the SHAP values of temperature and concentration indicated minimal contribution to the prediction (Fig. 11 B4–B5).

From cycle 4 onwards, the algorithm selected only one more experiment that used the best ligand up to this point (tBPh-CPhos). Evaluation of this experiment found a measured yield close to the predicted yield (cycle 3 prediction: 67%; actual yield in cycle 4: 77%). This improved prediction accuracy—as well as a general reduction in the uncertainty of the model (see Fig. 10 Cycle 4)—suggests that this area had been exploited sufficiently for EDBO to move on to explore other parts of the parameter space. The remaining experiments in cycle 4 explored new ligands whilst keeping the choice of base (KOAc) and solvent (DMAc) consistent with the base and solvent combination that had performed best up to that point (see Fig. 11 C).

Cycles 5 to 7 focused on exploiting the best ligand found in cycle 4 (CgMe-PPh) (see Fig. 11 D). The change in feature importance and strategy of EDBO was also observed to contrast sharply with the ligand SHAP values, which changed after cycle 4, as shown in Fig. 11 E. In particular, the ligand from cycles 1 to 3 seemed then to have a negative (blue) impact on the predicted yield, while the new ligand changed from little (white) to high (pink) impact.

**Fig. 13:**
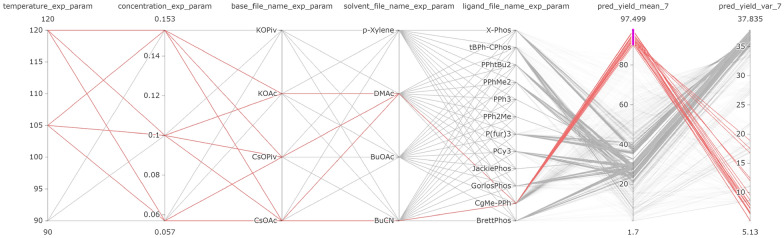
In the parallel coordinate plot, users can highlight the experimental conditions which, after the 7th cycle, the model predicts will achieve highest yield (’pred_yield_mean_7’)—in this case over 90%. This enables users to identify critical factors of RO **(T3)**

**Fig. 14:**
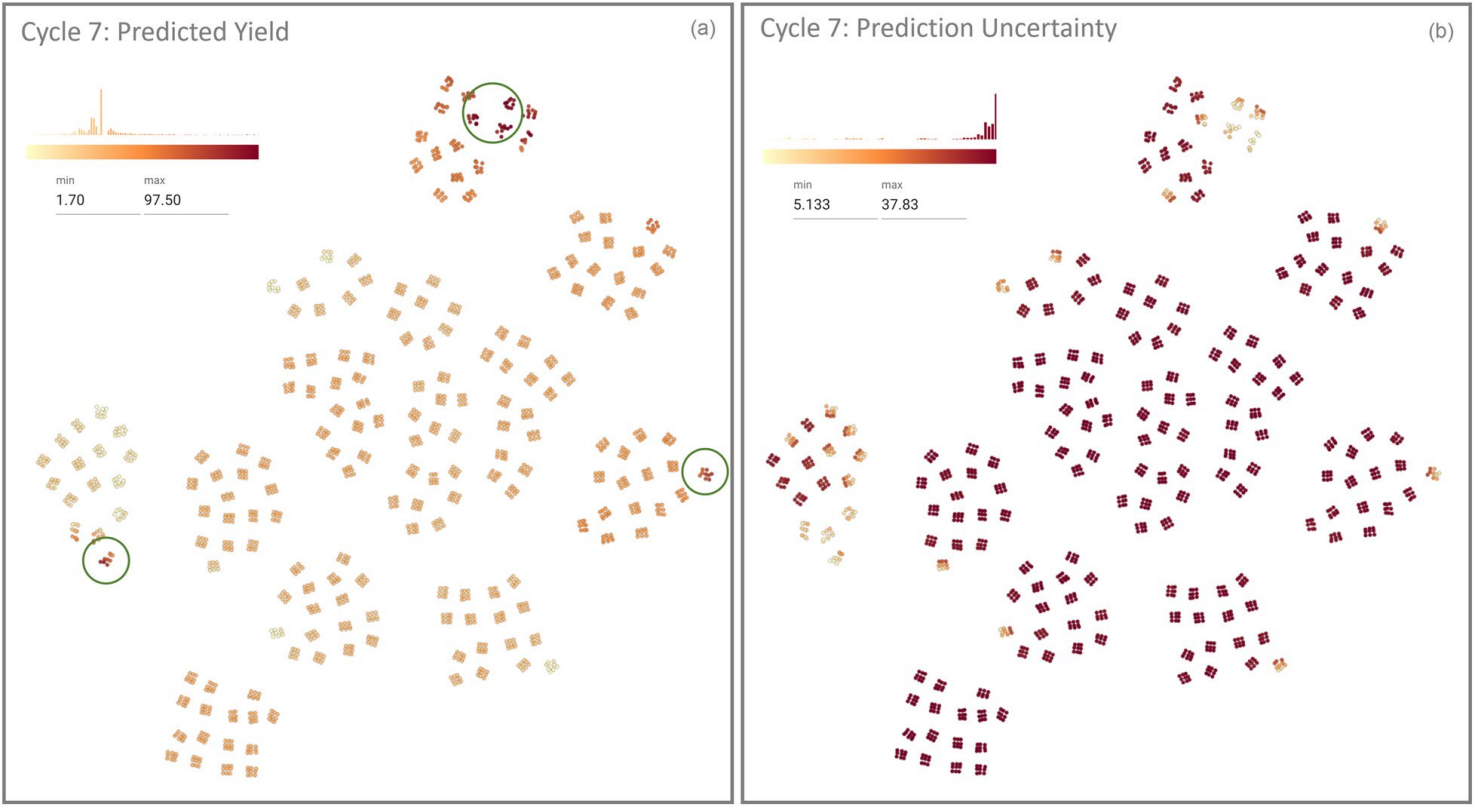
Projection view with all possible experimental points colored by their: **(a)** yield prediction and **(b)** prediction uncertainties after cycle 7. Potential local maxima are indicated by green circles

The optimization campaign was stopped after cycle 7 once an experiment had been found that achieved 100% yield. The chemists were not only interested in the best-performing experiment, but also in exploring all reactions predicted to give a high yield **(T3)**. To this end, the parallel coordinate feature of CIME4R and the PSE displaying the predicted yield after cycle 7 were utilized, as shown in Fig. 13 and Fig. 14. In this case, it was found that yields greater than 90% were predicted only for reactions using CgMe-PPh as the ligand with either DMAc or BuCN 7 as the solvent, with the base depending on the solvent selected. The numeric components were found to be less influential, and over 90% yield could be achieved with any of the given concentrations greater than 0.1 M and temperatures of 105° or higher, which suggests a robust range of operating conditions. In addition to the global optimum, local maxima in the parameter space, as shown in Fig. 14, might also be of interest. Visualizing the predicted yield for all possible experiments highlighted three maxima in the parameter space. This can be useful to know if the global maximum defines conditions that are operationally undesirable, for instance, if workup or isolation of the desired product becomes problematic.

### Case study 3: direct arylation reaction—human-AI collaboration to select the next experiments

**Fig. 15:**
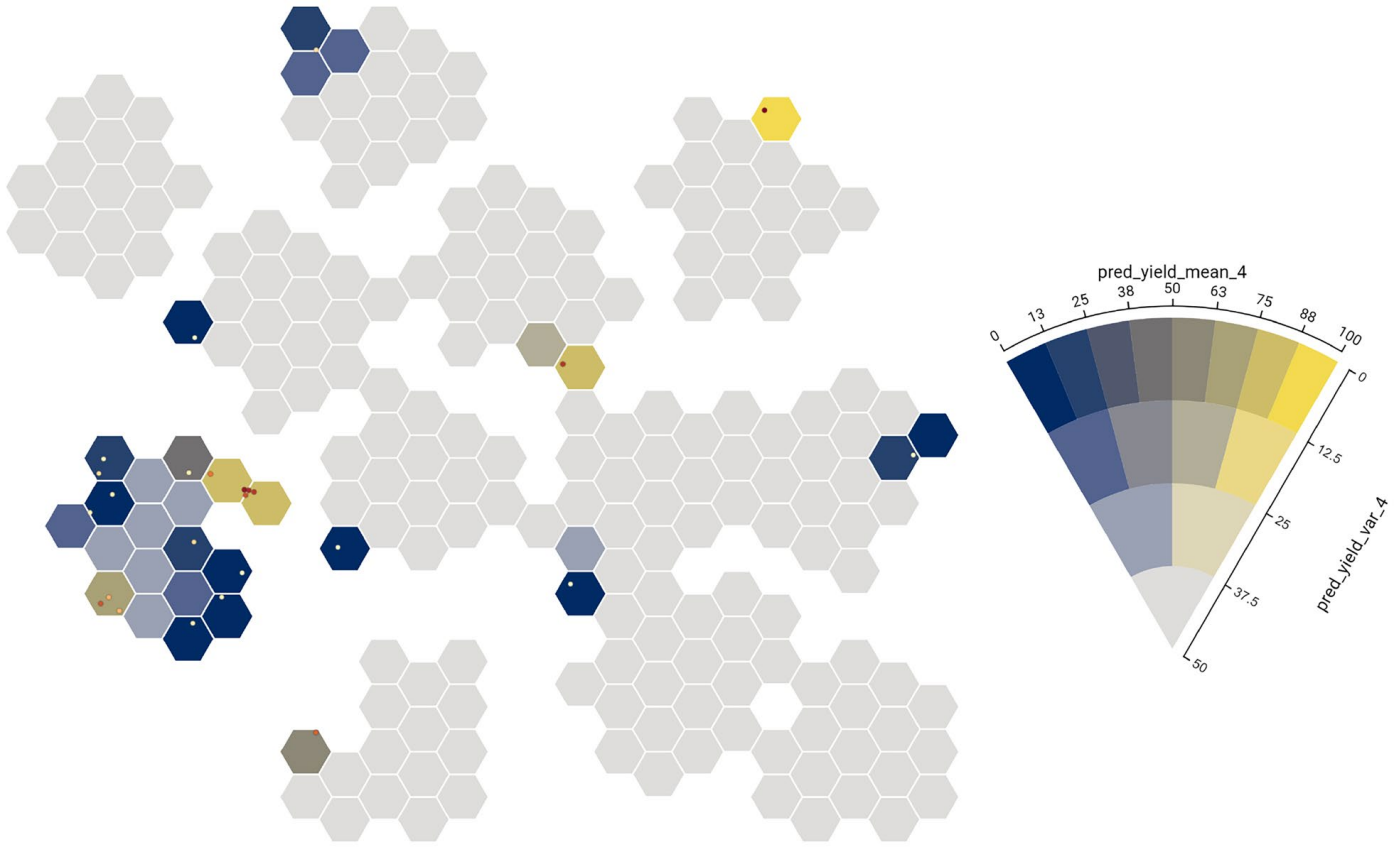
Projection of parameter space, aggregated by predicted yield and standard deviation after cycle 4 of the optimization process

**Fig. 16:**
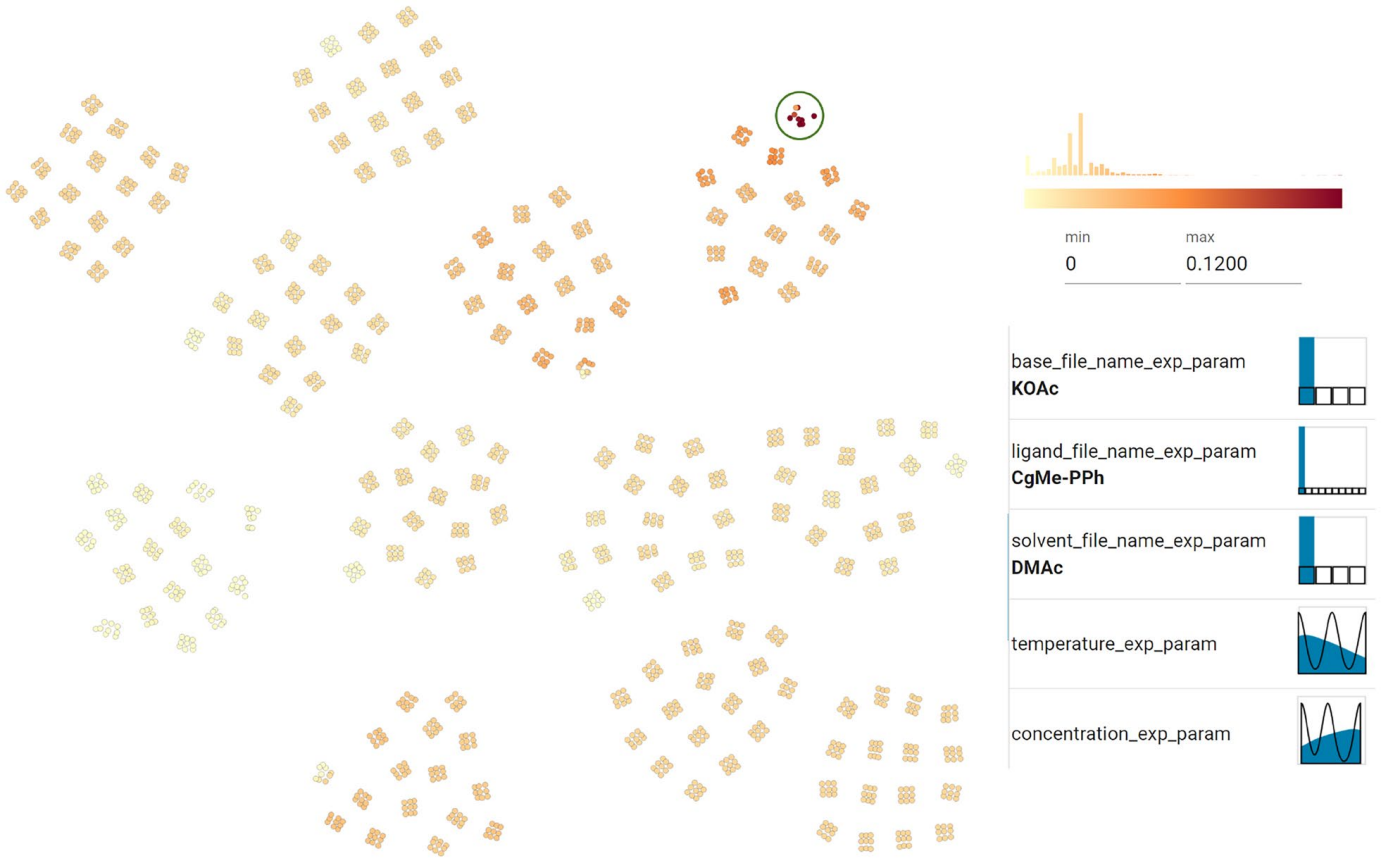
Parameter space encoded by the acquisition function after cycle 4 of the optimization process with a summary view of the four experiments with the highest acquisition function at this time

In the final case study, we tested to what extent CIME4R can facilitate the collaboration between humans and AI. For the same dataset as in case study 2, optimization was stopped after cycle 4, and we asked the chemists to decide on the next batch of five experiments using insights gained through CIME4R from EDBO and their own professional knowledge and intuition.

First, the chemists used the projection view to determine which areas of the parameter space had previously been explored, and the yield predictions (mean and standard deviation) from the latest model were displayed using the hex bins** (T1)**, as shown in Fig. 15. Three ligands were identified that had a high uncertainty in yield prediction, as no experiments had been performed in these areas; therefore, the chemists all selected to run at least one experiment using one of these unexplored ligands; for example, in one case JackiePhos was selected.

Then the chemists visualized the value of the acquisition function to see which experiment had the highest priority according to the EDBO algorithm and should thus be performed next to identify critical factors **(T3)**, as shown in Fig. 16. The highest values for the acquisition function were obtained for the most recent discovered optimal ligand (CgMe-PPh) and the previously best solvent/base combination: DMAc/KOAc. Since these experiments required only temperature and concentration to be varied, the chemists decided to take a sample of experiments from this area using temperature and concentration combinations that had not yet been studied under these conditions (e.g., a low temperature and high concentration). For the remaining experiments, the chemists stated they would decide between exploring other ligands that had not yet been explored or changing the base and solvent with the best ligand up to this point.

All chemists felt that they would have decided differently if they hadn’t used CIME4R. Without CIME4R, they would either have chosen all experiments based on their chemical intuition or run the experiments suggested by the EDBO algorithm. Comparison of the next batch of experiments chosen by the chemists to those selected by EDBO revealed some differences in terms of approach. While both the chemists and EDBO focused on exploiting the currently best ligand (CgMe-PPh), only the chemists explored alternative ligands that had not yet been used in the optimization campaign.

## Limitations and future work

As CIME4R is the first step towards better visualization and exploration of chemical reaction optimization campaigns, we envision an open-source community project that will improve and extend the functionality of CIME4R in the future. A major—and probably the greatest—limitation is the complexity of CIME4R. The learning curve is steep, and even after having been shown examples of all the tool’s features, users sometimes struggle to utilize CIME4R to its full potential. Currently, user guidance is implemented as comprehensive descriptions and hints for all functionalities directly in the tool. As part of future work, we will seek to reduce the steepness of the learning curve by applying best practices for how to onboard users (e.g., guided tours) and refining the visualizations such that they are unambiguous to the users (e.g., the difference visualizations seemed to have caused confusion about what it shows). We also plan to investigate how scientists use the tool in order to determine, which parts of the tool cause particular difficulties and which workflows could be automated to reduce users’ overhead. For example, in the case studies, we found that scientists frequently explored the iterations of the RO campaign. Considerable overhead is involved in adjusting all views to show one particular cycle of experiments. This could be optimized with a global time slider that automatically adapts all views to show the selected cycle.

Another limitation is the integration of CIME4R into existing infrastructure and workflows (i.e., how to feed the data into the tool). The data must be processed in a specific way to allow CIME4R to handle it properly. We plan to automate this process. Ideally, we would like to have an automated workflow that takes the data created in the experiments—optionally including additional data by an AI model—transforms it into the right format, and visualizes everything with CIME4R.

CIME4R has been tested on single-objective datasets only. The analysis of datasets with multiple objectives (e.g., optimizing for yield and temperature) is not yet properly supported. Although limited integration of multiple objectives in CIME4R would be feasible, more sophisticated approaches are necessary for their proper analysis [28].

To reduce memory usage, which is essential when dealing with large datasets, we had to implement strategies that come with the drawback of longer processing times. In the future, we will optimize the runtime of these processes wherever possible to reduce latency and make CIME4R more responsive when working with large datasets.

Recognizing the importance of analyzing dynamically generated data (i.e., iteratively adding new information), we plan to develop a function that allows this kind of data to be added to a dataset previously uploaded to CIME4R. For example, a dynamic change could be the addition of measurement data after a cycle has been finished. Another dynamic change could occur in the parameter space itself, for example, by adding parameters or changing parameter values.

In addition to potential improvements to CIME4R itself, there is also unexplored potential in the datasets generated for analysis in CIME4R. For example, one area of interest may include time-series data that arise from sampling a reaction at multiple time points. Further, aspects of a reaction’s work-up, isolation, and crystallization have not yet been explored in CIME4R. In relation to users understanding the optimization algorithms employed, other XAI features, such as SHAP interaction values, could be calculated for the dataset, and any parameters used for the optimization—for example, if the alpha parameter is varied in Bayesian optimization—could also be exported.

Finally, we evaluated CIME4R with three case studies. We decided on this approach because a quantitative study was not feasible at this point. Recruiting experts to participate in a study is often not easy and observational studies require a smaller number of participants [50] (in our case four experts). However, as we develop CIME4R further to better accustom user needs, we should also employ larger experimental studies where we evaluate CIME4R with qualitative and quantitative data.

## Conclusion

We have introduced CIME4R, an open-source, interactive tool that allows scientists to explore and understand chemical reaction optimization data. Users can navigate and analyze large, high-dimensional reaction parameter spaces to find high-performing experiments, understand the influence of various parameters on the objective, and make informed proposals for the next set of experiments. In the case of AI-guided reaction optimization, users can explore and thus better understand predictions made by an AI model. Scientists can combine this information with their own experience to make better decisions on the next experiments to be performed and thus gain from human-AI collaboration. We designed the tool to accommodate the tasks and challenges that reaction optimization data bring. In three case studies, we tested CIME4R’s usefulness in aiding domain experts with *(i)* understanding and learning from RO campaigns in retrospect, and *(ii)* human-AI collaborative decision-making. We found that domain experts successfully used CIME4R to complete the case studies and produced valuable insights from the datasets. Despite the steep learning curve involved, we are confident that scientists will benefit from using CIME4R to analyze their chemical RO data and that use of CIME4R will allow better integration of AI-guided RO.

## Supplementary Information

**Additional file 1.** Additional File 1 includes further details about the design and case studies of CIME4R.

## Data Availability

The datasets we used to test CIME4R and in the case studies are available online [32]. Project name: CIME4R—CIME for Reaction Optimization. Article project version: cime4rV0.1.6. Project home page: github.com/jku-vds-lab/reaction-cime. Demo website: reaction-optimization.jku-vds-lab.at. Demo video: www.youtube.com/watch?v=feqZWesf5Ws. Operating systems: Platform-independent. Programming language: TypeScript, python. Other requirements: the front end was tested on Chrome 119.0+ and Edge 119.0+; the back end requires python 3.10, RDKit 2020.09.5, gower 0.1.2, hdbscan 0.8.33, opentsne 1.0.0, umap-learn 0.5.3, and scikit-learn 1.2.2. License: BSD 3-Clause License.

## Abbreviations

AI: Artificial intelligence
CIME: ChemInformatics Model Explorer
CIME4R: CIME for reaction optimization
CSV: Comma-separated values
DoE: Design of experiments
DR: Dimensionality reduction
EDBO: Experimental design via Bayesian optimization
HTE: High-throughput experimentation
M: Molarity
MTBD: 7-Methyl-1,5,7-triazabicyclo[4.4.0]dec-5-ene
OFAT: One factor at a time
PCA: Principal component analysis
PSE: Projection Space Explorer
RO: Reaction optimization
SHAP: Shapley additive explanations
t-SNE: T-distributed stochastic neighbor embedding
UI: User interface
UMAP: Uniform manifold approximation and projection
VSUP: Value-suppressing uncertainty pallets
XAI: Explainable AI

## Footnotes

1 **UMAP:** Uniform Manifold Approximation and Projection [33]

2 **t-SNE:** t-Distributed Stochastic Neighbor Embedding [34]

3 **PCA:** Principal Component Analysis.

4 For diverging data (i.e., data with a meaningful center), such as SHAP values, we use a diverging color scale (i.e., a blue-white-red gradient) as shown in Fig. 3D

5 For example, columns that are part of time-series data should end with an underscore followed by a time step (e.g., acquisition_0, acquisition_1, and so on, containing the values of the acquisition function at cycle 0, 1, etc.).

6 The CSV file may be zipped before uploading to reduce the file size.

7 In accordance with Ahneman et al. [5], we use the potentially incorrect abbreviation BuCN, which we assume to refer to butyronitrile.
