# Supplementary material for: CIME4R: Exploring iterative, AI-guided chemical reaction optimization campaigns in their parameter space
Source: J Cheminform. 2024 May 10;16:51. doi: 10.1186/s13321-024-00840-1 (PMC11636728; doi:10.1186/s13321-024-00840-1)
Supplement: Supplementary file 1 — Additional file 1. Additional File 1 includes further details about the design and case studies of CIME4R. [file 13321_2024_840_MOESM1_ESM.pdf]

Supplementary Materials

CIME4R: Exploring iterative, AI-guided chemical reaction optimization campaigns in their parameter space

Christina Humer, Rachel Nicholls, Henry Heberle,
Moritz Heckmann, Michael Pühringer, Julius Hillenbrand, Thomas Wolf,
Maximilian Lübbesmeyer, Julian Heinrich, Giulio Volpin and Marc Streit

marc.streit@jku.at, christina.humer@jku.at,
giulio.volpin@bayer.com, julius.hillenbrand@bayer.com

December 20, 2023

1 Design

1.1 Projection view

Varying weight settings allows users to adapt the parameter space to best facilitate their analyses. For example, a scientist wants to give more weight to the solvent features because they suspect that this parameter carries the most information. Section 3 shows examples of projections performed with different weighting.

Figure 1 (right) shows the initial design of the aggregated projection view, where we visualized the projected parameter space by interpolating between the points. The interpolation visualization suggests continuity in the parameter space and the presence of data that is not found in the dataset.

Figure 2 shows the overview hex bin aggregation and a zoomed-in version (highlighted in red). The zooming is not an optical zoom (i.e., hexagons get bigger), but a semantic zoom, which means that the zoomed-in region shows more details about the parameter space (i.e., a higher number of bins within the same region than before).

Figure 3 shows an example of three juxtaposed projection views. The projection views show the predicted yield of a Bayesian RO on a deoxyfluorination reaction examined by Shields et al. [1] over three consecutive time steps. In the first experiment step, the model predictions have a high uncertainty due to the lack of available data. Over the next two experiment steps, the RO search space gets explored more and more, making the Bayesian optimization model more certain in its predictions. We can clearly see how more and more areas of the RO search space are explored and experiments with high yield are found (see Figure 3 step 3: yellow area).

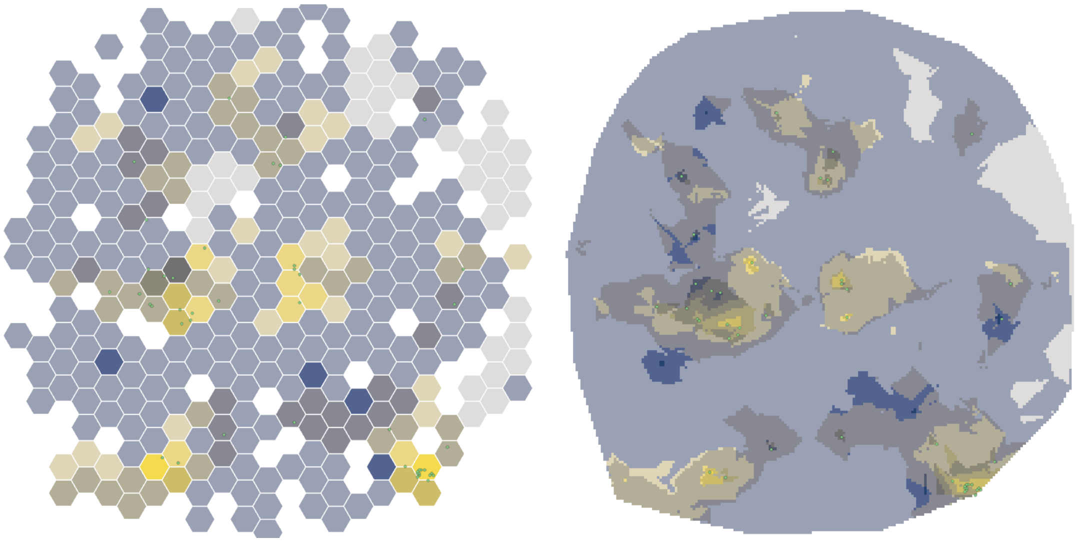

Figure 1: Aggregated view with a hexagonal binning of the RO space (left) compared to interpolating the RO space (right).

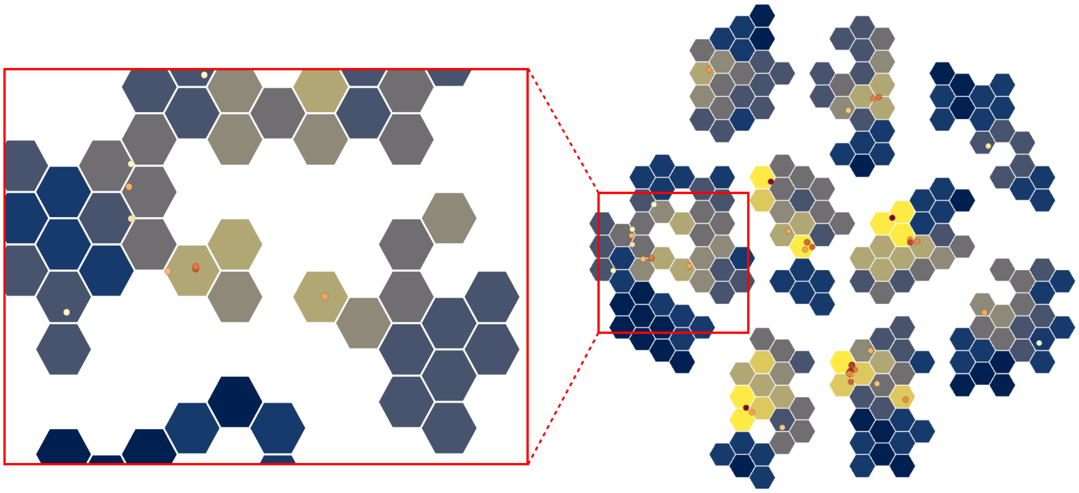

Figure 2: Example of semantic zoom of the hex bin aggregation.

2 Implementation

2.1 Projecting Data

For large datasets, we implemented an incremental version of PCA that processes the data in chunks before performing a final projection with t-SNE or UMAP. We checked the viability of this method by comparing projections with and without the use of chunking. One example is

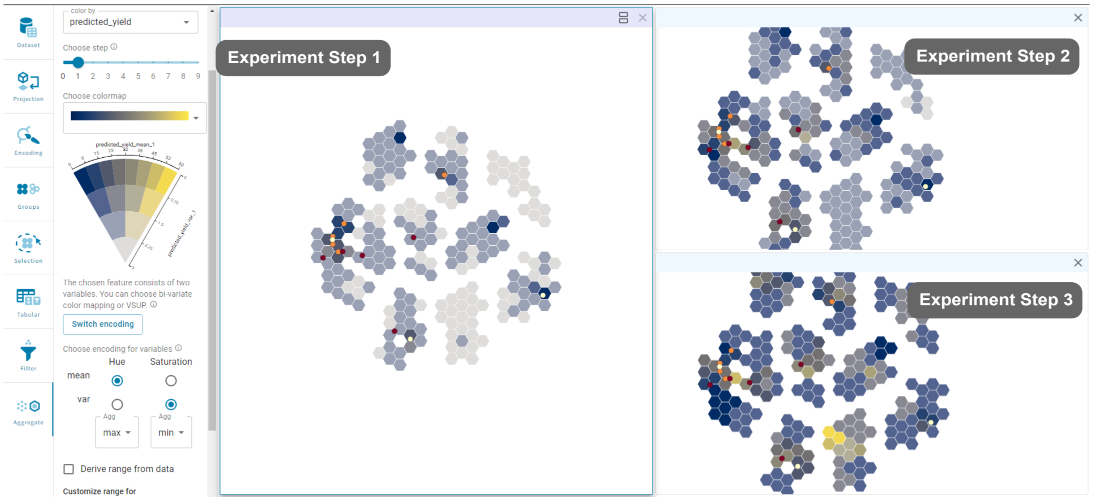

Figure 3: Example of juxtaposed projection views showing three steps of an RO workflow. This example shows the results of bayesian RO on deoxyfluorination [1].

shown in Figure 4, where we projected deoxyfluorination [1] reaction data *(left)* with t-SNE, and *(right)* with chunked t-SNE. For both projections, we used the substrate concentration, sulfonyl equivalent, base equivalent, and temperature experiment parameters and the sulfonyl, base, and solvent descriptor features. To activate the use of chunking for the same dataset, we started CIME4R with the "reaction_cime__max_memory_usage_for_projections=10" environment variable that limits the memory use of a calculating a projection to 10 MB (by default it is 50MB). The results of this projection can be seen in Figure 5, where the two projections show slight variations, but the overall layout of the two projections is the same.

3 Results

3.1 Case study 1

For projecting the data we selected the descriptors of the compounds. We used Gower's distance and assigned a weight of 1 to the aryl, base, and ligand descriptors, and a weight of 2 to the additive descriptors. We tried several different ways to project the dataset but found the setup mentioned before to create the most useful projection.

Descriptors of chemical compounds usually comprise a large number of numerical features. Without weighting, each descriptor feature would have the same influence on the projection as other features (see Figure 6 A). This would result in one or more of the descriptors outweighing other parameters chosen for the projection. By using weighted projection, all descriptors have the same importance, independent from the number of features a descriptor comprises of (see Figure 6 B).

Due to the large number of additives (22) investigated in this reaction, it can be challenging to get an overview of the most generalizable conditions. To aid with this, the aggregation feature of LineUp was utilized to group the experiments by the base and ligand, and the distribution of the measured yield is displayed, as seen in Figure 7. With this representation, it was observed that

t-SNE

```
reaction_cime.reaction_cime_api | Dataset bb508185-54a5-493b-b349-359ebfae5b39 has 5045 points.
reaction_cime.reaction_cime_api | Dataset requires ~41 MB of memory.
reaction_cime.reaction_cime_api | Starting preprocessing dataset bb508185-54a5-493b-b349-359ebfae5b39 with 300 columns at once...
reaction_cime.reaction_cime_api | Preprocessing columns 0 to 248...
reaction_cime.reaction_cime_api | Returning original data, because it fits in memory for downstream projections.
reaction_cime.reaction_cime_api | Featurizing rows 0 to 5045...
reaction_cime.reaction_cime_api | Calculating projection...
                   openTSNE.tsne | Precomputed initialization provided. Ignoring initalization-related parameters.
                   openTSNE.tsne | Automatically determined negative gradient method `bh`
reaction_cime.reaction_cime_api | Starting optimization
                   openTSNE.tsne | Automatically determined negative gradient method `bh`
reaction_cime.reaction_cime_api | Starting optimization
reaction_cime.reaction_cime_api | Save projection...
reaction_cime.reaction_cime_api | Finished!
```

chunked t-SNE

```
reaction_cime.reaction_cime_api | Dataset b3a0102f-5833-4ac4-ad31-f298b877d231 has 5045 points.
reaction_cime.reaction_cime_api | Dataset requires ~41 MB of memory.
reaction_cime.reaction_cime_api | Starting preprocessing dataset b3a0102f-5833-4ac4-ad31-f298b877d231 with 60 columns at once...
reaction_cime.reaction_cime_api | Preprocessing columns 0 to 60...
reaction_cime.reaction_cime_api | Preprocessing columns 60 to 120...
reaction_cime.reaction_cime_api | Preprocessing columns 120 to 180...
reaction_cime.reaction_cime_api | Preprocessing columns 180 to 240...
reaction_cime.reaction_cime_api | Preprocessing columns 240 to 248...
reaction_cime.reaction_cime_api | Using 49 PCA components to fit in memory
reaction_cime.reaction_cime_api | Featurizing rows 0 to 1223...
reaction_cime.reaction_cime_api | Fitting incremental PCA for rows 0 to 1223...
reaction_cime.reaction_cime_api | Featurizing rows 1223 to 2446...
reaction_cime.reaction_cime_api | Fitting incremental PCA for rows 1223 to 2446...
reaction_cime.reaction_cime_api | Featurizing rows 2446 to 3669...
reaction_cime.reaction_cime_api | Fitting incremental PCA for rows 2446 to 3669...
reaction_cime.reaction_cime_api | Featurizing rows 3669 to 4892...
reaction_cime.reaction_cime_api | Fitting incremental PCA for rows 3669 to 4892...
reaction_cime.reaction_cime_api | Featurizing rows 4892 to 5045...
reaction_cime.reaction_cime_api | Fitting incremental PCA for rows 4892 to 5045...
reaction_cime.reaction_cime_api | Featurizing rows 0 to 1223...
reaction_cime.reaction_cime_api | PCA transform rows 0 to 1223...
reaction_cime.reaction_cime_api | Featurizing rows 1223 to 2446...
reaction_cime.reaction_cime_api | PCA transform rows 1223 to 2446...
reaction_cime.reaction_cime_api | Featurizing rows 2446 to 3669...
reaction_cime.reaction_cime_api | PCA transform rows 2446 to 3669...
reaction_cime.reaction_cime_api | Featurizing rows 3669 to 4892...
reaction_cime.reaction_cime_api | PCA transform rows 3669 to 4892...
reaction_cime.reaction_cime_api | Featurizing rows 4892 to 5045...
reaction_cime.reaction_cime_api | PCA transform rows 4892 to 5045...
reaction_cime.reaction_cime_api | Calculating projection...
                   openTSNE.tsne | Precomputed initialization provided. Ignoring initalization-related parameters.
                   openTSNE.tsne | Automatically determined negative gradient method `bh`
reaction_cime.reaction_cime_api | Starting optimization
                   openTSNE.tsne | Automatically determined negative gradient method `bh`
reaction_cime.reaction_cime_api | Starting optimization
reaction_cime.reaction_cime_api | Save projection...
reaction_cime.reaction_cime_api | Finished!
```

Figure 4: Example t-SNE projection log output of deoxyfluorination [1] reaction data with (upper) and without (lower) chunking.

using MTBD as the base with one of three ligands gave the highest yields on average.

3.2 Case study 2

For projecting the data we selected *(i)* the experimental parameters, *(ii)* the DFT descriptors for each chemical compound, and *(iii)* and the shap values for each component at the end of the final (7th cycle). We used the Gower distance metric and assigned a weight of 1 to each category of features except for the ligand descriptors, to which we assigned a total weighting of 3. The weighting was necessary mainly due to the descriptors but also to account for the importance of the ligand component in this reaction.

The need for weighting can also be seen when using categorical factors when descriptors are not present. For example in Figure 8 the projection was generated using t-SNE with Gower metric and a weighting of 1 for all experimental parameters. Here the main clusters represent experiments

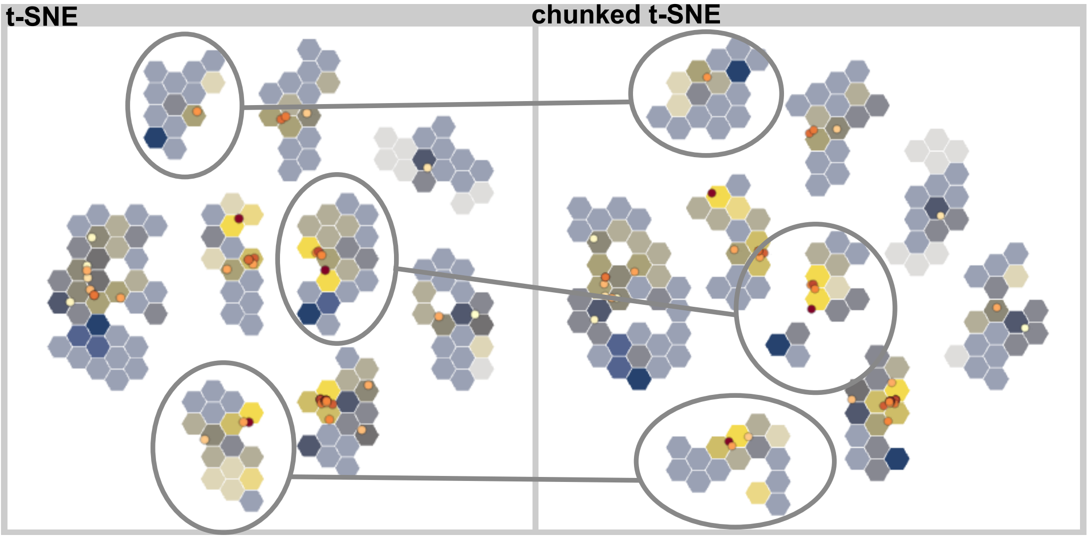

Figure 5: Example t-SNE projection view of deoxyfluorination [1] reaction data with (left) and without (right) chunking. The layout of the projection is slightly different, but the overall structure and cluster formation are the same.

that were performed using a combination of one base and one solvent, whilst the different ligands studied are clustered together in the smallest sub-cluster. While this overview may provide a good visualization for the effects of the base and solvent, it is not helpful if the effect of the ligand is of most interest. Therefore having the ability to increase the weighting of each factor, is important to enable the user to visualize the data in multiple meaningful ways. For example, in Figure 9, the projection was repeated with the weighting of the ligand set to 2. This improved the distribution of the points according to ligand, enabling an overview of the best conditions between ligands to be compared.

Changing the projection can also be helpful for understanding the reaction optimization campaign. In Figure 10 an overview of the parameter space, where each cluster represents the combination of one base and one solvent (like in Figure 8) is shown, with aggregation of the predicted yield and prediction standard deviation across each cycle. Here, it is easier to visualize how the model's prediction varies with each solvent and base combination, compared to the projection used in the main paper. However, for simplicity, in the main paper, we show only one projection which was found to be the most useful.

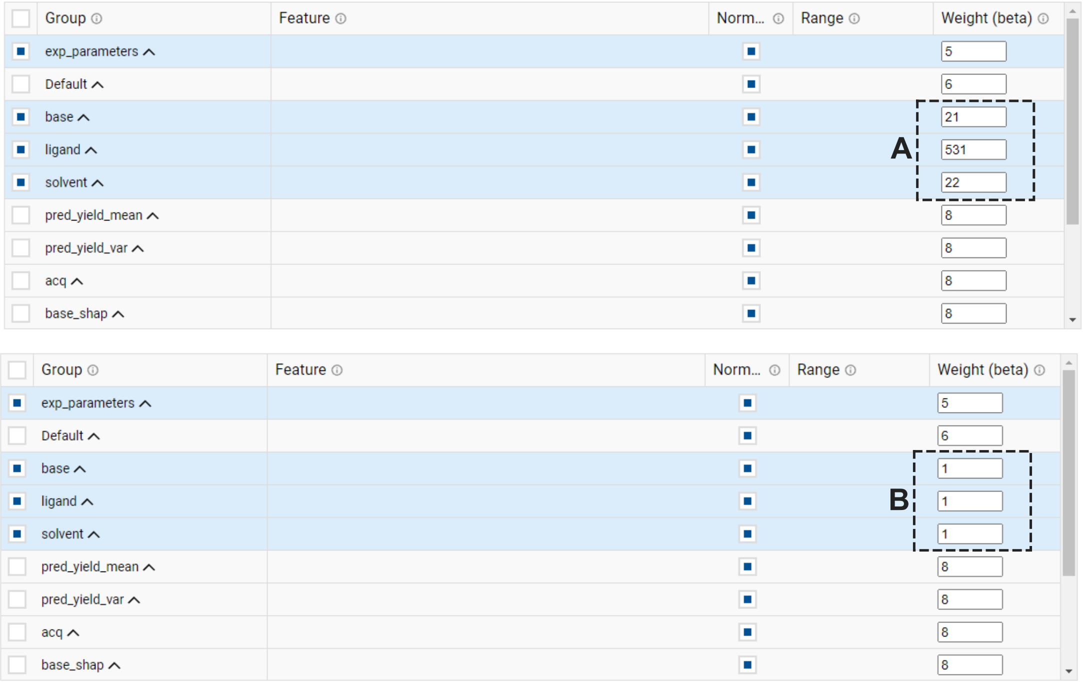

Figure 6: Example of a projection (A) without and (B) with weighting. (A) In this example the projection includes the five experiment parameters and the descriptors for the base (21 features), ligand (531 features), and solvent (22 features). Each parameter as well as each descriptor feature has the same influence on the projection. This also means that the ligand descriptor has a total relative importance of 531, while the base, solvent, and temperature only have 22, 21, and 1 relative importance respectively during the projection. (B) In this example, we assigned a total weight of 1 to each descriptor. Therefore each descriptor feature only has a weighting of $1/n$ where n is the number of features a descriptor comprises of (e.g., a weight of 1/531 for each ligand descriptor feature). Overall this results in a balanced importance for the projection, meaning that the descriptors and experiment parameters each have a total weight of 1.

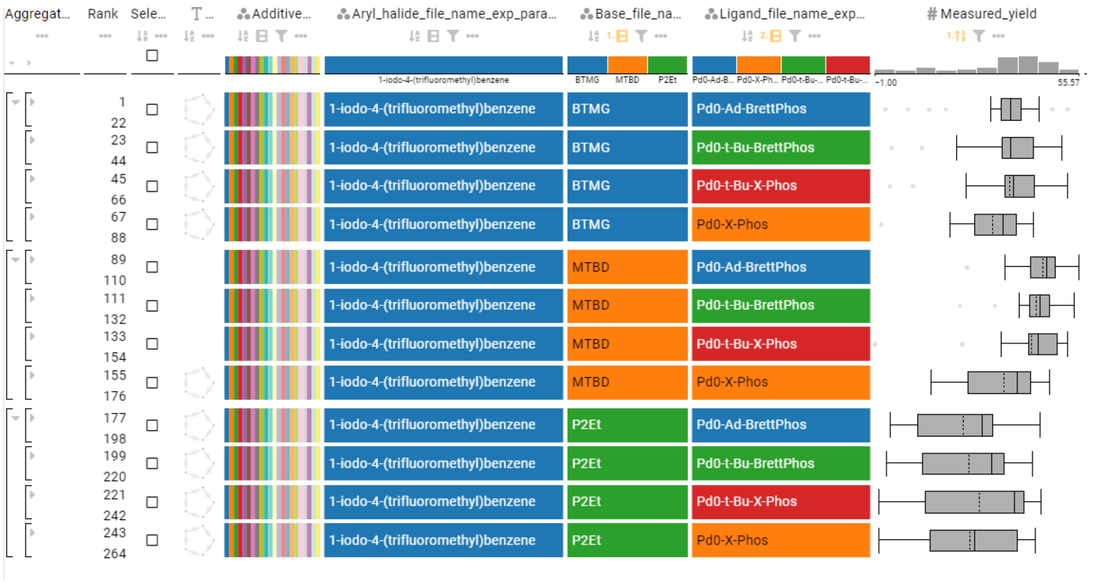

Figure 7: Aggregation feature of LineUp utilized to group all experiments performed with the Aryl Iodide substrate by the base and ligand to visualize the distribution of the measured yield

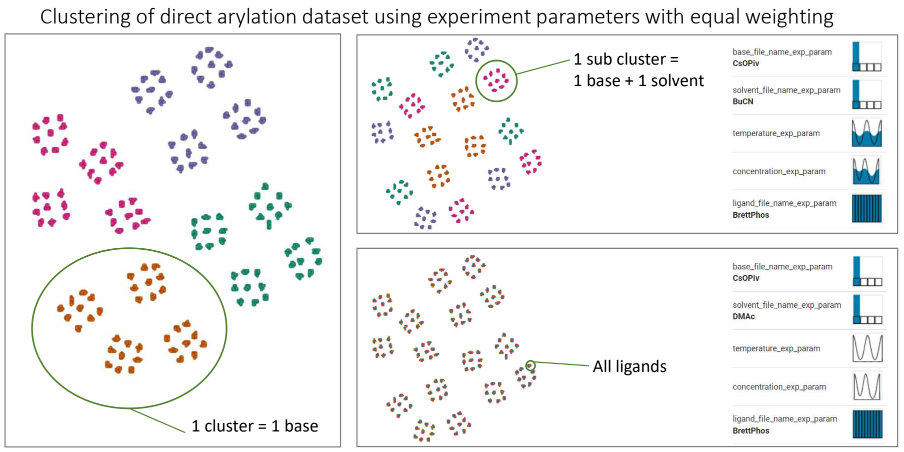

Figure 8: Comparison of weighted projection with all experiment parameters weighted at 1.

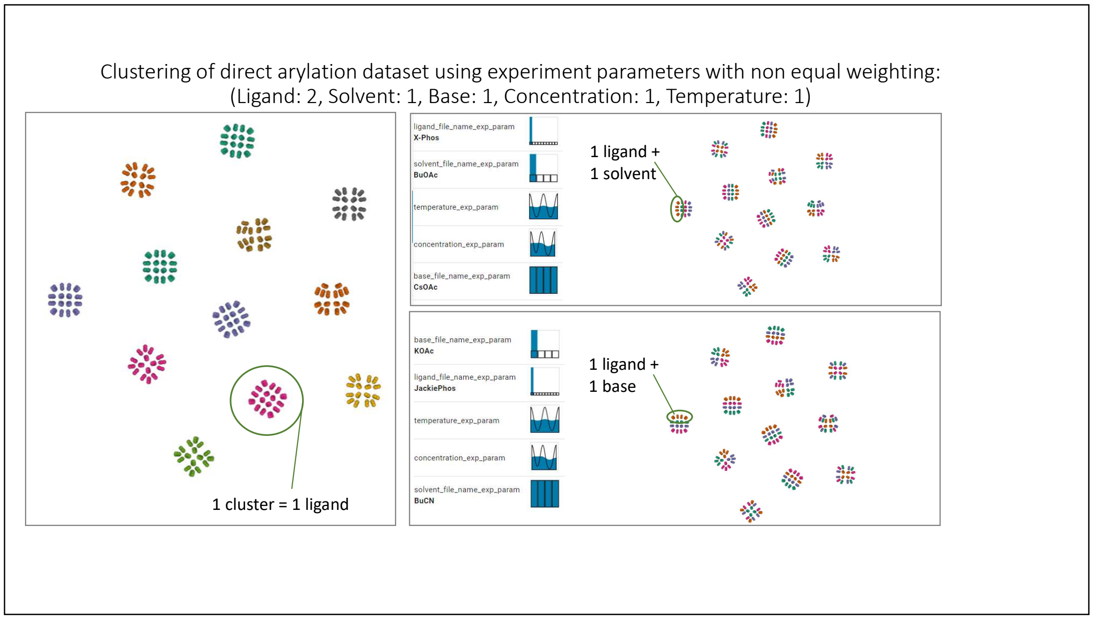

Figure 9: Comparison of weighted projection with all experiment parameters weighted at 1 except for ligand at weight 2.

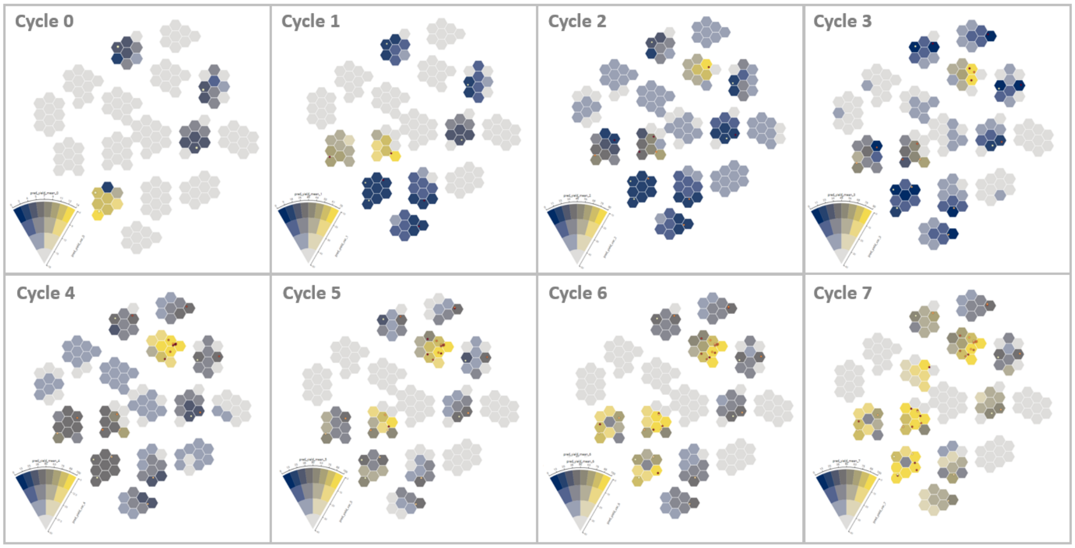

Figure 10: Alternative projection of parameter space, aggregated by predicted yield and standard deviation showing the progression of EDBO after each cycle.

References

[1] Shields, B.J., Stevens, J., Li, J., Parasram, M., Damani, F., Alvarado, J.I.M., Janey, J.M., Adams, R.P., Doyle, A.G.: Bayesian reaction optimization as a tool for chemical synthesis. Nature **590**(7844), 89–96 (2021). doi:10.1038/s41586-021-03213-y. Number: 7844 Publisher: Nature Publishing Group. Accessed 2021-04-16
